# Triggering ubiquitination of IFNAR1 protects tissues from inflammatory injury

**DOI:** 10.1002/emmm.201303236

**Published:** 2014-01-31

**Authors:** Sabyasachi Bhattacharya, Kanstantsin V Katlinski, Maximilian Reichert, Shigetsugu Takano, Angela Brice, Bin Zhao, Qiujing Yu, Hui Zheng, Christopher J Carbone, Yuliya V Katlinskaya, N Adrian Leu, Kelly A McCorkell, Satish Srinivasan, Melanie Girondo, Hallgeir Rui, Michael J May, Narayan G Avadhani, Anil K Rustgi, Serge Y Fuchs

**Affiliations:** 1 Department of Animal Biology, School of Veterinary Medicine, University of PennsylvaniaPhiladelphia, PA, USA; 2 Division of Gastroenterology, Department of Medicine and Abramson Cancer Center, University of PennsylvaniaPhiladelphia, PA, USA; 3 Department of Genetics, Perelman School of Medicine, University of PennsylvaniaPhiladelphia, PA, USA; 4 Department of Pathobiology, School of Veterinary Medicine, University of PennsylvaniaPhiladelphia, PA, USA; 5 Department of Cancer Biology, Kimmel Cancer Center, Thomas Jefferson UniversityPhiladelphia, PA, USA

**Keywords:** hepatitis, inflammation, interferon, pancreatitis, receptor

## Abstract

Type 1 interferons (IFN) protect the host against viruses by engaging a cognate receptor (consisting of IFNAR1/IFNAR2 chains) and inducing downstream signaling and gene expression. However, inflammatory stimuli can trigger IFNAR1 ubiquitination and downregulation thereby attenuating IFN effects *in vitro*. The significance of this paradoxical regulation is unknown. Presented here results demonstrate that inability to stimulate IFNAR1 ubiquitination in the *Ifnar1^SA^* knock-in mice renders them highly susceptible to numerous inflammatory syndromes including acute and chronic pancreatitis, and autoimmune and toxic hepatitis. *Ifnar1^SA^* mice (or their bone marrow-receiving wild type animals) display persistent immune infiltration of inflamed tissues, extensive damage and gravely inadequate tissue regeneration. Pharmacologic stimulation of IFNAR1 ubiquitination is protective against from toxic hepatitis and fulminant generalized inflammation in wild type but not *Ifnar1^SA^* mice. These results suggest that endogenous mechanisms that trigger IFNAR1 ubiquitination for limiting the inflammation-induced tissue damage can be purposely mimicked for therapeutic benefits.

**Subject Categories** Immunology; Digestive System

## Introduction

Inflammation has evolved as an adaptive process that combines the counteractive elements of tissue destruction versus healing to limit and eventually eliminate the harmful effects of irritants and infectious agents. Initial stages of inflammation that exacerbate tissue damage are often followed by subsequent regeneration/repair of tissue to restore its function (Medzhitov, 2008). Failure to progress to these later stages contributes to development of chronic pathologic conditions associated with numerous human diseases including pancreatitis and hepatitis, arthritis, and neurodegenerative syndromes. Despite the enormous medical significance of persistent inflammation, the mechanisms that restrain the tissue damage and ensure the transition to the restorative phase largely remain to be understood (Nathan ' Ding, 2010).

Chronic viral infections and growing tumors are among known inducers of persistent inflammation (Li *et al*, 2006). Cytokines that belong to the family of type 1 interferons (IFN) protect the host against viruses and tumors (Stark *et al*, 1998). IFN act on cells through engaging a cell surface-located cognate receptor (formed by IFNAR1 and IFNAR2 chains) and subsequent stimulation of the signal transduction cascade including activation of the Janus kinases (JAK) and signal transduction and activators of transcription (STAT1/2) proteins (Uze *et al*, 2007). The latter interact with IRF9 to form a specific transcriptionally active complex capable of inducing a plethora of IFN-stimulated proteins that act in concert to mediate the anti-viral, anti-tumorigenic and immunomodulatory effects of IFNs. These effects underlie the use of pharmacologically formulated IFN for treatment of viral infections, cancers and multiple sclerosis (Stark *et al*, 1998; Platanias, 2005; Stark ' Darnell, 2012).

However, the benefits of IFN actions to the host are offset by tissue toxicities elicited by these cytokines (Trinchieri, 2010). Importantly, while anti-viral effects of IFN action could be achieved at low concentrations, a higher dose is required to achieve the anti-proliferative and anti-survival effects of IFN on tumor and benign cells (Jaitin *et al*, 2006). Furthermore, the toxic effects of IFN correlate with its affinity for IFNAR1 (Jaitin *et al*, 2006; Kalie *et al*, 2007; Thomas *et al*, 2011; de Weerd *et al*, 2013) and require a much higher cell surface density of its receptor (Moraga *et al*, 2009; Levin *et al*, 2011; Piehler *et al*, 2012). The cell surface levels of IFN receptor and ensuing sensitivity of cells to IFN are negatively regulated by the ubiquitination, endocytosis and degradation of IFNAR1 facilitated by the β-Trcp E3 ubiquitin ligase (Kumar *et al*, 2003, 2007). The ability of β-Trcp to interact with IFNAR1 and to ubiquitinate this protein requires phosphorylation on specific serine residues (Ser526 in mouse IFNAR1 or Ser535 in human receptor) within a conserved degron motif located in the intracellular domain of IFNAR1 (Kumar *et al*, 2003, 2004).

This IFNAR1 phosphorylation triggered by IFN (Kumar *et al*, 2004; Marijanovic *et al*, 2006) through JAK-mediated activation of protein kinase D2 (PKD2) attenuates IFN signaling by ubiquitination-driven downregulation and degradation of IFNAR1 (Zheng *et al*, 2011a,c2011c). Importantly, this phosphorylation of IFNAR1 can be induced by ligand-unrelated stimuli that altogether desensitize cells to IFN (reviewed in (Fuchs, 2013). For example, PKD2-activating vascular endothelial growth factor (VEGF) can trigger ubiquitination and downregulation of IFNAR1 and blunt the cellular responses to IFN (Zheng *et al*, 2011b). Other stimuli capable of IFN-independent phosphorylation of IFNAR1 utilize a different pathway (Liu *et al*, 2008, 2009b) that involves IFNAR1 Ser526/535 phosphorylation by casein kinase 1α (Liu *et al*, 2009a) amplified by an adjacent priming phosphorylation (Bhattacharya *et al*, 2010), which is mediated by the p38 stress-activated protein kinase (Bhattacharya *et al*, 2011). These stimuli include inflammatory cytokines such as interleukin-1β and tumor necrosis factor-α [IL1β and TNFα (Huangfu *et al*, 2012)], and bacterial lipopolysaccharide [LPS, (Qian *et al*, 2011)].

The inflammatory stimuli trigger IFNAR1 phosphorylation, ubiquitination and degradation and, as a result, largely deprive cells from ability to respond to their future encounter with IFN *in vitro* (Qian *et al*, 2011; Zheng *et al*, 2011b; Huangfu *et al*, 2012). Should this regulation occur *in vivo*, it might pose a medical problem of reduced efficacy of pharmaceutical IFN in inflamed tissues. Furthermore, the very existence of such mechanism is somewhat counterintuitive for the host defense as it may limit the anti-viral and anti-tumorigenic benefits of endogenously produced IFN within the regions of inflammation.

Hence, we sought to investigate the manifestation and biological significance of inflammation-induced IFNAR1 ubiquitination and downregulation *in vivo*. We initially focused on the role of IFN signaling and IFNAR1 stability in development of acute pancreatitis, a deadly disease affecting millions of people worldwide [reviewed in (Pandol, 2006)]. Whereas the etiology of this disease is complex, common pathogenetic mechanisms include food/alcohol-induced pancreatic hyperstimulation and pancreatic duct obstruction leading to increased pancreatic duct pressure and reflux of active trypsin. As a result, trypsin and other proteolytic enzymes become activated within pancreatic acinar cells resulting in their autolysis and induction of local inflammation [reviewed in (Pandol, 2006; Reichert ' Rustgi, 2011)]. Intriguingly, pancreatitis induces many pathways known to downregulate IFNAR1 such as VEGF (Kuehn *et al*, 1999), unfolded protein response (Kubisch ' Logsdon, 2007), and pathogen recognition receptors (Pastor *et al*, 2004; Li *et al*, 2005). Furthermore, a number of reported cases of iatrogenic pancreatitis have been attributed to the IFN-based drugs (Trivedi ' Pitchumoni, 2005), and IFN-induced pancreatic inflammation was observed in mice injected with poly I:C (Hayashi *et al*, 2011).

Our current data suggest that stimulation of IFNAR1 downregulation during pancreatitis functions as a protective mechanism that limits the extent of tissue damage and promotes the transition of inflammatory process to the stages of tissue regeneration. This mechanism also plays an important role in hepatic and generalized inflammatory syndromes. We further demonstrate that pharmacologic induction of PKD2 and p38 kinases aimed to preemptively accelerate downregulation of IFNAR1 reduces the severity of inflammatory tissue injury and may prove useful in treatment of acute inflammatory conditions.

## Results

### Important role of IFN in pathogenesis of acute pancreatitis

**Figure 1:**
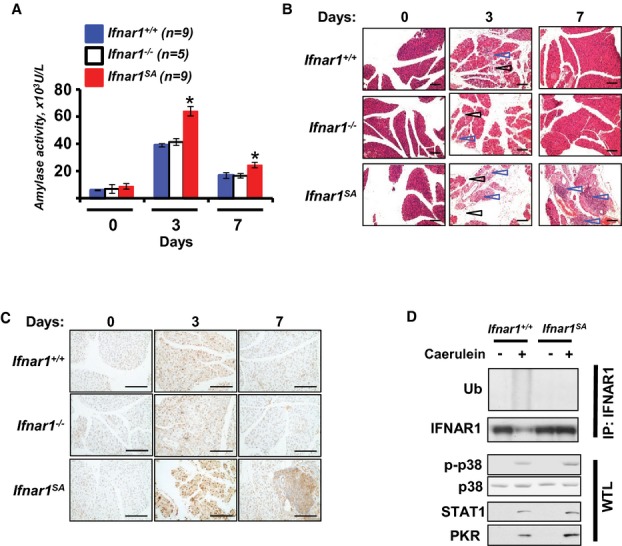
Acute pancreatitis is exacerbated in Ifnar1SA mice incapable of stimulating IFNAR1 ubiquitination.
Experimental pancreatitis induced after injection of caerulein assessed by amylase activity levels in plasma from indicated mice at indicated time points; (*P < 0.05 compared to *Ifnar1*^*+/+*^).H'E staining of pancreata obtained from mice at the indicated times after caerulein injections. Black and blue arrows point to the regions of acinar degeneration and leukocyte infiltration (respectively). The size bar (here and thereafter) is 200 μm. Additional pictures from other animals in similar or greater magnification as well as quantification of acinar tissue loss are shown in Supplementary Figs 3, 4 and 5.IHC analysis of activated p-p38 kinase in pancreata from panel B.Immunoblotting analyses of IFNAR1 immunoprecipitated from the whole pancreatic tissue lysates from indicated mice harvested at 2 days after caerulein or saline injections was carried out using the indicated antibodies. Analyses of activated p38 kinase as well as STAT1 and PKR proteins in whole tissue lysates (WTL) are also shown. Total levels of p38 kinase are depicted as a loading control. Source data are available for this figure.

To delineate the role of IFN in pancreatitis we used a clinically relevant model involving injections of caerulein peptide that mimics the effects of cholecystokinin and induces key pathogenetic elements of acute pancreatitis including pancreatic duct obstruction and premature activation of trypsin (Pandol, 2006; Reichert *et al*, 2013). Caerulein injections triggered elevated plasma levels of IFNβ (Supplementary Fig 1) and pancreatic mRNA levels of IFN-stimulated genes such as *Isg15*, *Irf7* and *Stat1* (Supplementary Fig 2). Furthermore, this treatment led to a noticeable increase in the levels of amylase in blood plasma (Fig 1A) and in characteristic histopathologic alterations in the pancreas such as primary pancreatic tissue autodigestive injury (manifested by the loss of acinar cells) and evidence of secondary inflammation including activation of p38 kinase, tissue edema and immune cell infiltration (Fig 1B–D, Supplementary Figs 3–5).

Remarkably, wild type (*Ifnar1*^*+/+*^) and *Ifnar1*^*−/−*^ mice displayed a similar severity of these alterations (Fig 1, Supplementary Figs 3–5) suggesting that either IFN signaling plays no role in pathogenesis of acute pancreatitis or IFNAR1 is inactivated under these conditions in wild type tissues. Levels of IFNAR1 protein in the pancreas were indeed decreased after caerulein treatment (Fig 1D). Given that trypsin (activated in the inflamed pancreas) can cleave the extracellular domain of IFNAR1 *in vitro* (Supplementary Fig 6) and diverse inflammatory stimuli can induce ubiquitination of the intracellular domain of IFNAR1 leading to its endocytosis and degradation in cultured mammalian cells (Qian *et al*, 2011; Zheng *et al*, 2011b; Huangfu *et al*, 2012), it is plausible that a similar proteolytic inactivation of IFNAR1 could occur *in vivo*.

Indeed, we observed that induction of pancreatitis robustly increased plasma concentration of several inflammatory cytokines including IL1α (2.6-fold), IL1β (12.8-fold), VEGF (70.5-fold) TNFα (4.5-fold), and IL6 (585.2-fold) as well as stimulated p38 kinase activation in pancreatic tissue (Fig 1C,D). Furthermore, analysis of lysates from pancreatic tissues from wild type mice revealed an increase in ubiquitination of IFNAR1 in response to caerulein injections (Fig 1D). Given these data, it is plausible that the role of IFN in pathogenesis of pancreatitis is masked by rapid elimination of IFNAR1 protein in wild type animals.

To test this possibility, we took advantage of the fact that numerous pathways leading to ubiquitination of IFNAR1 in response to inflammatory stimuli converge on phosphorylation of IFNAR1 on Ser526/Ser535 that enables the recruitment of β-Trcp E3 ligase (Huangfu ' Fuchs, 2010; Fuchs, 2013). We previously developed the knock-in mice harboring a single *Ifnar1*^*S526A*^ allele (Liu *et al*, 2009b; Zheng *et al*, 2011b); all tissues of these animals expressed the IFNAR1^S526A^ mutant protein resistant to ubiquitination induced by VEGF or LPS *in vitro* (Qian *et al*, 2011; Zheng *et al*, 2011b). Here we used animals homozygous for this mutant allele (*Ifnar1*^*SA*^ mice). Importantly, naïve *Ifnar1*^*SA*^ mice developed normally and exhibit neither any signs of growth retardation nor gross abnormalities (Supplementary Fig 7), nor hallmarks of constitutive pancreatic inflammation (Fig 1B,C). Cells from these animals exhibited a slower rate of IFNAR1 degradation yet did not display a hyper-reactive IFN signaling as evident from the lack of constitutive activation of STAT1 (Supplementary Fig 8), sustained ability of their hematopoietic cells to engraft and reconstitute bone marrow in lethally irradiated mice (Material and Methods and experiments described below), normal blood cell counts and serum chemistry profiles as well mitochondrial activities in the peripheral tissues similar to that of wild type mice (Supplemental Table 1).

However, under conditions of experimental pancreatitis, *Ifnar1*^*SA*^ mice exhibited noticeably higher pancreatic tissue levels of IFN-stimulated proteins (STAT1 and PKR, Fig 1D) and IFN-stimulated genes such as *Irf7* (Supplementary Fig 2) compared with wild type animals. Consistent with the key role of *Irf7* in subsequent IFN induction (Platanias, 2005; Uze *et al*, 2007; Stark ' Darnell, 2012), levels of IFNβ in plasma of caerulein-treated *Ifnar1*^*SA*^ mice were also elevated (Supplementary Fig 1). Importantly, while the IFNAR1^S526A^ mutant receptor remained sensitive to the proteolytic effects of extracellular trypsin *in vitro* (Supplementary Fig 6), we did not observe its ubiquitination and downregulation in response to the caerulein-induced pancreatitis *in vivo* (Fig 1D). These results demonstrate that analysis of inflammation in *Ifnar1*^*SA*^ mice can provide us with a tool to differentiate between IFNAR1 ubiquitination-dependent and –independent mechanisms.

**Figure 2:**
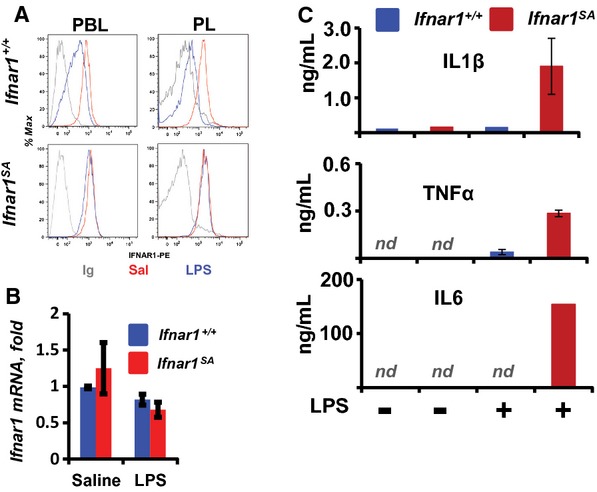
Inflammatory stimuli fail to downregulate IFNAR1 in the *Ifnar1^SA^* mice.
FACS analysis of the cell surface IFNAR1 levels in peripheral blood leukocytes (PBL) or peritoneal leukocytes (PL) from indicated mice harvested at 3 h after injection of either saline (Sal, red) or LPS (blue). Ig, antibody isotype control (gray).Analysis of relative *Ifnar1* mRNA levels in PBL treated as in panel A (*n* = 3 for each group).ELISA analyses of the levels of indicated cytokines (in ng/ml) in blood plasma of indicated mice (*n* = 3 for each genotype) treated with saline or LPS for 12 h; *nd*, levels below 40 pg/ml.

To determine whether induction of IFNAR1 ubiquitination/downregulation is common under diverse inflammatory conditions, we utilized the LPS treatment to stimulate generalized inflammation *in vivo*. Injection of LPS decreased the cell surface IFNAR1 protein levels in leukocytes from wild type but not *Ifnar1*^*SA*^ mice (Fig 2A). Given that mRNA levels of Ifnar1 were not dramatically affected (Fig 2B), this result suggests that either inflammation-induced downregulation of IFNAR1 *in vivo* is ubiquitination-dependent or *Ifnar1*^*SA*^ mice do not efficiently produce inflammatory cytokines or IFN in response to LPS. Contrary to the latter hypothesis, much higher levels of IL1β, TNFα, IL6 and IFNβ were detected in plasma from *Ifnar1*^*SA*^ than from wild type animals treated with LPS (Fig 2C, Supplementary Fig 9). These data collectively indicate that promoting the ubiquitination of IFNAR1 is important for the downregulation of this receptor in response to local and systemic inflammatory stimuli *in vivo*.

Remarkably, in the context of acute pancreatic inflammation, *Ifnar1*^*SA*^ mice exhibited an increased amylase levels compared to the wild type animals (Fig 1A). A greater severity of pancreatitis in these mice was also manifested by an extensive acinar cell loss, pronounced leukocyte infiltration and robust activation of p38 kinase (Fig 1B,C and Supplementary Figs 3–5). Together with a greater expression of IFN-stimulated genes mRNA and proteins (Supplementary Fig 2, Fig 1D) these results indicate that stimulating IFNAR1 ubiquitination and attenuating IFN signaling may have a protective function to curb pancreatic inflammation. They also argue that IFN signaling plays an important role in development of acute pancreatitis.

### Stimulation of IFNAR1 ubiquitination modulates specific leukocyte recruitment and enables pancreatic tissue repair

**Figure 3:**
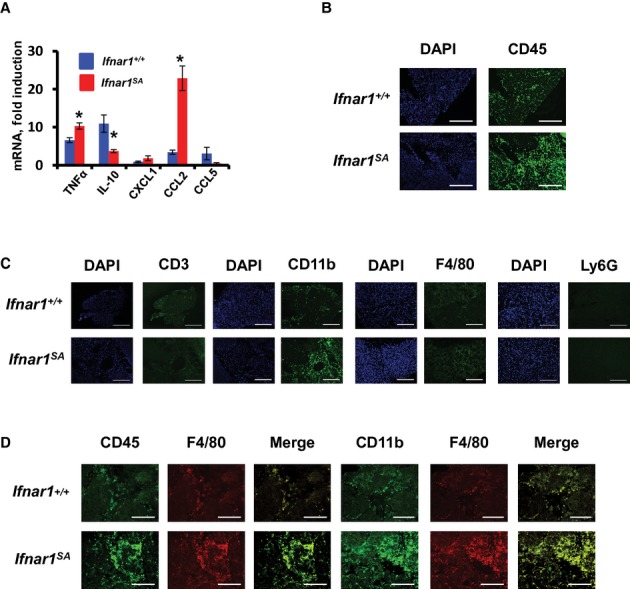
An aberrant cytokine balance and inflammatory monocyte recruitment to inflamed pancreatic tissues from *Ifnar1^SA^* mice.
Fold induction of mRNA of *TNF*α, *IL10*, *CXCL1*, *CCL2* and *CCL5* (normalized per β*-actin* mRNA) in pancreata from indicated mice (*n* = 3 for each genotype) harvested 3 days after caerulein injections. Normalized mRNA levels in mice that received saline were assigned a value of 1.0. *P < 0.05 in comparison with wild type mice.Immunofluorescent analysis of pancreata from indicated mice (harvested 7 days after caerulein injections) was carried out on frozen sections using anti-CD45 antibody.Immunofluorescent analysis of indicated pancreata processed as in B using indicated antibodies. Positive and negative controls for the specificity of these antibodies are shown in Supplementary Fig 10.Immunofluorescent co-localization analysis of indicated pancreata processed as described in B.

We next aimed to investigate the mechanisms by which the inflammation-stimulated ubiquitination of IFNAR1 may contribute to protection of pancreatic tissues from excessive injury. We first focused on a balance between inflammatory cytokines (e.g. TNFα) and anti-inflammatory cytokines (e.g. IL10). Noticeably higher *TNF*α mRNA levels were found in pancreatic tissues from *Ifnar1*^*SA*^ mice than in their wild type counterparts (Fig 3A). In addition, the pancreatitis-induced increase in blood plasma TNFα levels were also greater in *Ifnar1*^*SA*^ (7.89 fold) than in *Ifnar1*^*+/+*^ (4.50-fold) mice. Levels of IL10 protein in blood plasma were higher in caerulein-treated *Ifnar1*^*SA*^ animals (251 ± 2 versus 114 ± 2 pg/ml in wild type mice) indicative of systemic anti-inflammatory response and consistent with reported increase of this cytokine in caerulein-treated mice (Zhou *et al*, 2012) and documented ability of IFN to induce IL10 production in leukocytes *in vitro* (Aman *et al*, 1996; Rudick *et al*, 1996). However, unexpectedly low *IL10* mRNA levels were found in the pancreatic tissues from *Ifnar1*^*SA*^ animals (Fig 3A). This result suggests that downregulation of IFNAR1 in cells localized in the focus of inflammation restrains production of inflammatory TNFα while promoting the expression of anti-inflammatory IL10.

Given that inflammatory cytokines in the pancreas can be produced by infiltrating leukocytes, we then assessed the recruitment of these cells to the inflamed tissues in wild type and *Ifnar1*^*SA*^ mice. Upon caerulein injections, the pancreata from *Ifnar1*^*SA*^ animals displayed a greater extent of infiltration by leukocytes as seen from histologic analysis (Fig 1B and Supplementary Fig 3) and immunofluorescent analysis of CD45^+^ cells (Fig 3B). Among these leukocytes, the CD11b^+^ myeloid cells displayed a prominent preference for the diffuse pancreatic infiltration in the *Ifnar1*^*SA*^ mice (Fig 3C and control staining in Supplementary Fig 10A). In addition, CD11b+/F4/80+ cells represented a majority of leukocytes in the highly infiltrated areas in these mice (Supplementary Fig 10B). Pancreata of these mice also exhibited a low expression of mRNA for RANTES/CCL5) chemotactic for T cells (Fig 3A) and, accordingly, noticeably fewer CD3^+^ T cells (Fig 3C). Conversely, a robust increase in expression of a monocyte-attracting chemokine MCP-1/CCL2 and relatively low levels of neutrophil chemoattractant CXCL1 (Fig 3A) was consistent with predominant pancreatic infiltration with monocytes/maturing macrophages (CD11b^+;^ F4/80^+^; Ly6G^low^) in *Ifnar1*^*SA*^ mice (Fig 3C,D, Supplementary Figs 10B and 11).

**Figure 4:**
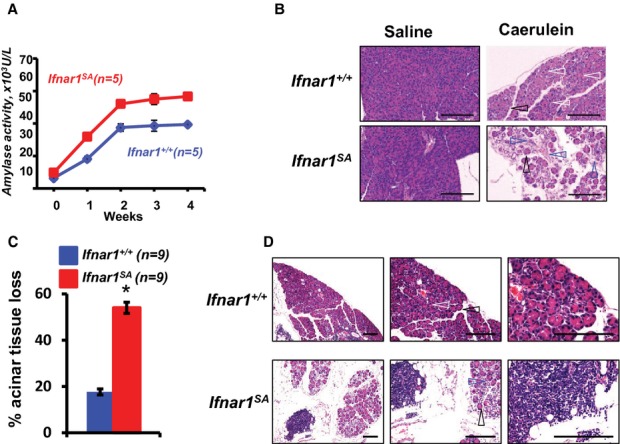
Inability to stimulate IFNAR1 ubiquitination augments and prolongs tissue damage and inhibits tissue regeneration under conditions of chronic pancreatitis.
Amylase activity in serum from indicated mice injected with caerulein (50 μg/kg, 5 days a week for indicated amount of weeks).H'E staining of pancreata from mice that received injections of saline or caerulein for 4 weeks as described in panel A. Arrows point to the areas of restored tissue (white) and chronic tissue damage manifested in continuous edema (black), and fibrotic tissue replacing acinar epithelium (blue).Acinar tissue damage in indicated pancreata from mice described in panel B was quantified as% of acinar tissue loss compared to saline-treated animals. *P < 0.05.H'E staining of pancreata from indicated caerulein-treated mice (treated as in panel B) is depicted at different magnification to highlight tissue degeneration and leukocytic infiltrates. Arrows indicate areas described in panel B.

A careful analysis of kinetics of acute pancreatitis in wild type mice shows that tissue damage peaks at Day 3 after caerulein injection whereas pancreatic glands collected at Day 7 shows the signs of subsided inflammation and reestablishment of tissue integrity. Yet, pancreata from *Ifnar1*^*SA*^ mice exhibited very few sings of acinar cell restoration and displayed a persistent immune infiltration (Fig 1B and Supplementary Fig 3). An even greater increase in pancreatic inflammation and damage was observed in *Ifnar1*^*SA*^ mice receiving multiple injections of caerulein to model chronic pancreatits (Fig 4A–D and Supplementary Fig 12). Unlike wild type mice that displayed both signs of inflammation (including edema and leukocytic infiltration) and restored tissue (Fig 4B), pancreata from *Ifnar1*^*SA*^ animals remained highly inflamed (Fig 4B,D and Supplementary Fig 12) and exhibited pronounced signs of tissue damage, degeneration and fibrosis (Fig 4B–D). These data strongly suggest that induction of IFNAR1 ubiquitination restricts the extent of inflammation and tissue injury and enables pancreatic tissue regeneration in chronic pancreatitis.

### Autoimmune and toxic hepatitis is exacerbated by inefficient ubiquitination of IFNAR1

**Figure 5:**
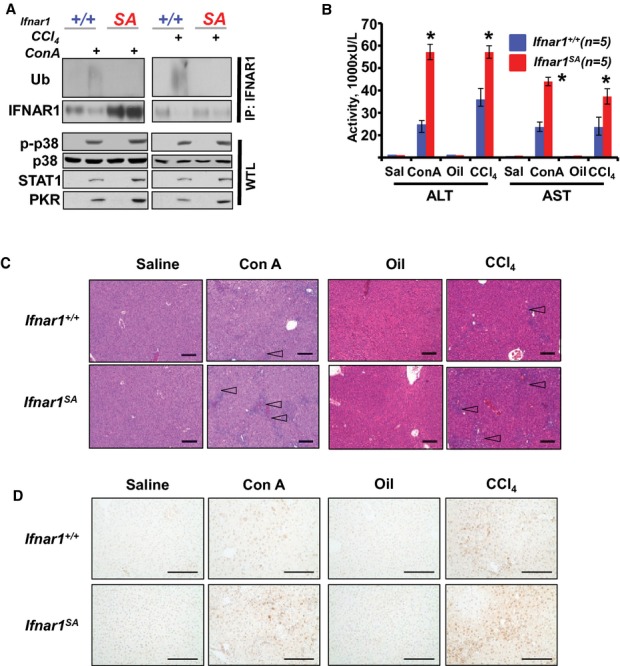
Deficient ubiquitination of IFNAR1 sensitizes mice to experimental autoimmune and toxic hepatitis.
Immunoblotting analyses of IFNAR1 immunoprecipitated from the liver lysates from indicated mice harvested at 10 h after treatment with ConA (or saline as a control) or 48 h after treatment with CCl_4_ (or oil as a control) was carried out using the indicated antibodies. Analyses of activated p38 kinase as well as STAT1 and PKR proteins in whole tissue lysates (WTL) are also shown. Total levels of p38 kinase are depicted as a loading control.Activity of alanine (ALT) and aspartate (AST) aminotransferases in blood plasma from wild type or *Ifnar1^SA^* mice (*n* = 5 for each group) treated as indicated. *P < 0.05 compared to wild type.H'E staining of liver tissue from mice described in panel B. Arrows point to hepatic lesions and concurrent areas of leukocyte infiltration.IHC analysis of activated p-p38 kinase in liver tissues from panel C. Source data are available for this figure.

We sought to further examine whether ubiquitin-mediated downregulation of IFNAR1 could be important for limiting the damage to other inflamed tissues. To this end, we utilized two independent models of experimental hepatitis induced either by concanavalin A [ConA, that triggers autoimmune hepatitis (Tiegs *et al*, 1992)] or carbon tetrachloride [CCl_4_, that causes hepatic necrosis and reactive hepatitis (Luster *et al*, 2001)]. Remarkably, in both of these models we observed a robust induction of IFNAR1 ubiquitination and downregulation of this protein (but not mRNA) in liver tissues from wild type but not *Ifnar1*^*SA*^ mice (Fig 5A, Supplementary Fig 13). Conversely, the latter animals exhibited a noticeably higher expression of STAT1 and PKR proteins (Fig 5A), expression of *TNF*α mRNA (Supplementary Fig 14) and levels of plasma IFNβ (Supplementary Fig 15 and data not shown). In parallel, the severity of hepatic inflammation was prominently greater in *Ifnar1*^*SA*^ mice compared to the wild type animals as judged by increased release of liver aminotransferases from hepatocytes into the bloodstream (serum AST and ALT, Fig 5B).

Furthermore, livers harvested from *Ifnar1*^*SA*^ mice exhibited dramatic histopathologic alterations (Fig 5C and Supplementary Fig 16) along with robust activation of p38 kinase (Fig 5D), greater number of hepatic lesions and consistently higher hepatitis grade (Table 1). These alterations in *Ifnar1*^*SA*^ mice together with a more pronounced leukocyte infiltration (Supplementary Fig 17) strongly suggest that ubiquitination of IFNAR1 plays a protective role during hepatitis of either autoimmune or toxic etiology. Collectively, these data implicate ubiquitination and degradation of IFNAR1 as an important common mechanism that limits the extent of inflammatory liver injury.

**Table 1:** Number of hepatic lesions per field and histopathologic grade of hepatitis (in parenthesis) scored in tissues from indicated mice treated as indicated

Genotype/BMT	+/+	SA	+/+ → SA	SA → +/+
Treatment	No. of lesions (grade)	No. of lesions (grade)	No. of lesions (grade)	No. of lesions (grade)
Saline	0.0 ± 0.0 (0)	0.0 ± 0.0 (0)	ND	ND
ConA	2.2 ± 1.3 (2)	7.0 ± 1.2 (3)*	ND	ND
Oil	0.0 ± 0.0 (0)	0.0 ± 0.0 (0)	0.0 ± 0.0 (0)	0.0 ± 0.0 (0)
CCl_4_	1.8 ± 0.8 (2)	6.4 ± 0.9 (3)*	1.6 ± 0.6 (2)	5.8 ± 0.9 (3)*

* *P* < 0.05 compared to +/+; ND, not determined.

### Downregulation of IFNAR1 on bone marrow-derived cells determines the severity of inflammatory syndromes

**Figure 6:**
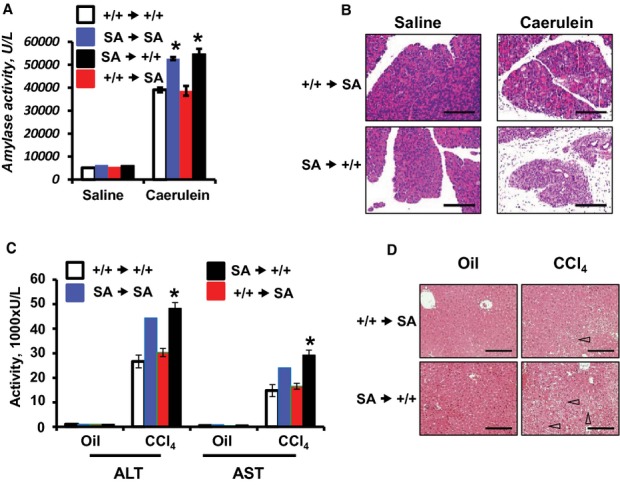
Elimination of IFNAR1 in bone marrow-derived cells protects inflamed tissues from excessive damage.
Amylase activity levels in serum from chimeric mice after indicated bone marrow transplantation (donor recipient, *n* = 4 for each group) following caerulein injections (*P < 0.05). +/+, *Ifnar1*^*+/+*^; SA, *Ifnar1^SA^*.H'E staining of pancreata obtained from chimeric mice described in panel A.Activity of alanine (ALT) and aspartate (AST) aminotransferases from serum of chimeric mice after indicated bone marrow transplantation (donor recipient, *n* = 3 for each group) and injection of CCl_4_ or oil (as a vehicle control). *P < 0.05.H'E staining of liver tissue from mice described in panel C.

Within the foci of inflammation, an accelerated ubiquitin-dependent turnover of IFNAR1 may occur on diverse type of cells including parenchymal, endothelial and stromal cells as well as on immune cells recruited to the target organs. To differentiate between the roles of IFNAR1 ubiquitination in hematopoietic/immune compartment versus peripheral tissues, the bone marrow transfer (BMT) approach was utilized. We initially compared the severity of acute pancreatitis in *Ifnar1*^*SA*^ animals that received BMT from either mice of the same genotype or from wild type animals. The latter chimeric mice challenged with caerulein exhibited a lesser increase in the levels of serum amylase activity (Fig 6A, blue bars v. red bars). Conversely, the replacement of bone marrow in wild type mice by that from *Ifnar1*^*SA*^ animals augmented the induction of serum amylase (Fig 6A, white bars v. black bars). These data were further corroborated by the histopathologic analyses (Fig 6B and Supplementary Fig 18) demonstrating that severity of pancreatitis correlated with expression of ubiquitination-deficient *Ifnar1*^*SA*^ mutant in the bone marrow-derived cells but not in the peripheral tissues.

The importance of IFNAR1 ubiquitination within the central immune compartment for tissue protection was next investigated in the hepatitis model induced by CCl_4_. Chimeric animals that received BMT from wild type mice consistently exhibited a lesser serum level of liver aminotransferases (Fig 6C), fewer hepatic lesions and overall lower hepatitis scores (Fig 6D, Supplementary Fig 19 and Table 1). On the other hand, experiments using the reverse approach (BMT from *Ifnar1*^*SA*^ animals into the wild type mice) revealed a robustly augmented liver tissue damage and inflammatory phenotype (Fig 6C,D, Supplementary Fig 19 and Table 1). These data indicate that ubiquitination and degradation of IFNAR1 in the bone marrow-derived cells recruited to the foci of inflammation plays a common major tissue protective role.

### Pharmacologically induced ubiquitination and downregulation of IFNAR1 protects from toxic hepatitis and fulminant generalized inflammation

If downregulation of IFNAR1 as a byproduct of inflammation limits tissue injury, a preemptive pharmacologic elimination of this receptor might be used as a tissue protective approach. Agents triggering this elimination should theoretically be capable of inducing IFNAR1 serine phosphorylation and subsequent ubiquitination. An endogenous lipid mediator, lysophosphatidic acid (LPA) was previously shown to act on immune cells (Graler ' Goetzl, 2002) and activate both PKD2 and p38 kinases (Chiu *et al*, 2007). Thus, we tested the effects of LPA on IFNAR1 ubiquitination and related inflammatory conditions.

**Figure 7:**
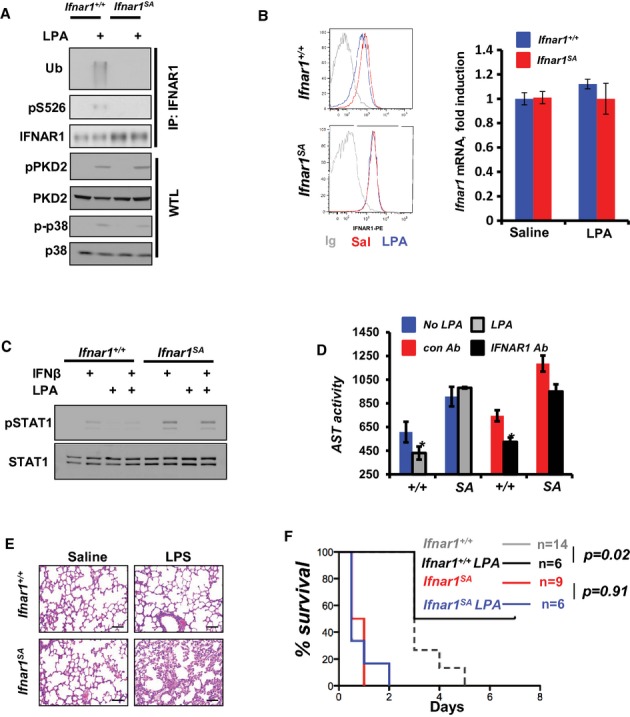
Pharmacologic induction of IFNAR1 ubiquitination attenuates the severity of toxic hepatitis and septic shock.
Immunoblotting analyses of immunoprecipitated IFNAR1 and whole tissue lysates (WTL) from the spleens of mice injected or not with LPA (10 mg/kg; 50 min prior to harvesting) was carried out using the indicated antibodies.FACS analysis of IFNAR1 cell surface levels in splenocytes from indicated mice harvested at 3 h after treatment of either saline or LPA (20 μg/ml). Right panel depicts relative *Ifnar1* mRNA levels analyzed under these conditions (*n* = 3 for each group).Immunoblotting analysis of STAT1 phosphorylation and levels in splenocytes from *Ifna1r*^*+/+*^ and *Ifnar1^SA^* mice treated for 3 h with either saline or LPA (20 μg/ml) and then isolated and treated *in vitro* with IFNβ (1000 U/ml for 30 min) where indicated.AST activity in plasma from *Ifnar1*^*+/+*^ or *Ifnar1^SA^* mice collected 24 h after the injection of acetaminophen (APAP, 150 mg/kg, i.p.). Where indicated, mice were also injected with LPA (i.v., 10 mg/kg, *n* = 4 mice for treatment and control groups) or anti-IFNAR1 neutralizing antibody (i.v., 100 μg, *n* = 3 mice for treatment and control groups) at 0, 8 and 16 h after APAP treatment).H'E staining of lungs from indicated mice treated with saline or LPS for 12 h.Kaplan-Meier analysis of survival of indicated mice treated with LPS (10.5 mg/kg) without or with LPA (10 mg/kg). Source data are available for this figure.

To this end, the *in vivo* signaling experiments utilizing the intravenous injection of mice with LPA followed by analysis of IFNAR1 in the splenic tissues were used. LPA treatment induced phosphorylation of IFNAR1 on Ser526 in wild type mice (Fig 7A). As expected, this effect was not seen in *Ifnar1*^*SA*^ animals whose receptor contains the alanine residue in this position. Importantly, wild type but not *Ifnar1*^*SA*^ tissues exhibited an increase in ubiquitination of IFNAR1 (Fig 7A). Furthermore, injection of wild type mice with LPA led to a noticeable downregulation of IFNAR1 protein (but not mRNA) on splenocytes (Fig 7B) and attenuated the sensitivity of these cells to IFN *in vitro* as assessed by IFN-induced activation of STAT1 (Fig 7C). Importantly, neither of these events was observed in *Ifnar1*^*SA*^ tissues (Fig 7B,C). These data indicate that LPA can be utilized to trigger IFNAR1 ubiquitination and degradation and to attenuate IFN signaling *in vivo*. Moreover, the *Ifnar1*^*SA*^ mice deficient in IFNAR1 ubiquitination yet competent in LPA-induced activation of PKD2 and p38 kinases (Fig 7A) serve as a critical control that allows to distinguish the role of LPA-induced IFNAR1 elimination from IFNAR1-independent pleiotropic effects of LPA.

We tested the effects of LPA in a clinically relevant model of toxic hepatitis induced by analgesic agent Acetaminophen (APAP), whose overdose causes severe liver damage and greatly contributes to acute liver failure of non-viral etiology in human patients (Ostapowicz ' Lee, 2000; Bernal *et al*, 2010). A greater extent of APAP-induced liver damage (assessed by the plasma aminotransferase levels) was detected in *Ifnar1*^*SA*^ mice compared to wild type animals. While AST/ALT levels in APAP-treated wild type were significantly decreased upon treatment with LPA, we did not observe this effect in *Ifnar1*^*SA*^ mice (Fig 7D and Supplementary Fig 20). Given that the latter mice express receptor whose ubiquitination and levels are not affected by LPA (Fig 7A,B), this result specifically suggests that LPA-induced ubiquitination and degradation of IFNAR1 can protect hepatic tissues from toxic injury.

To further determine the benefit of targeting IFNAR1 in inflammation, we used a neutralizing antibody against IFNAR1 that binds to its extracellular domain (Sheehan *et al*, 2006) and, therefore, should block signaling from both wild type and ubiquitination-deficient receptor. Indeed, injection of this antibody noticeably decreased APAP-induced hepatitis in all animals (Fig 7D and Supplementary Fig 21). Furthermore, similar results with either LPA or anti-IFNAR1 antibody were obtained when hepatotoxicity was induced using CCl_4_ (Supplementary Figs 22 and 23). Collectively, these results suggest that targeting IFNAR1 via either blocking its extracellular domain (by antibody) or inducing ubiquitination of its intracellular domain (by LPA-like agents) can be used to protect liver against excessive inflammatory damage.

Intriguingly, the ability of LPA to moderate generalized inflammation induced by LPS has been previously reported, although the mechanisms underlying these effects are yet to be understood (Fan *et al*, 2008; Zhao *et al*, 2011). While toxicity of LPS was attenuated upon genetic ablation of *Ifnar1* (Karaghiosoff *et al*, 2003), it remains unclear whether induction of IFNAR1 ubiquitination in wild type animals might function as a mechanism for reducing the LPS toxicity or/and a target for protective effects of LPA. While conducting the experiments on LPS-induced cytokines (Fig 2) we were unable to compare the levels of these cytokines between wild type and *Ifnar1*^*SA*^ mice past 24 h after LPS treatment because of early lethality in the *Ifnar1*^*SA*^ group. This lethality was likely associated with a strikingly greater extent of lung injury including pronounced leukocyte infiltration of the interstitial and alveolar spaces, edema and alveolar distortion observed within 12 h after LPS injection (Fig 7E). Remarkably, a detailed analysis of survival revealed a significant protective effect of LPA treatment in wild type but not in *Ifnar1*^*SA*^ mice (Fig 7F). These results indicate that ubiquitination of IFNAR1 is an intrinsic mechanism that plays a critical role in preventing the fulminant course of LPS-induced septic shock. Furthermore, collectively these data provide a proof of principle for pharmacological stimulation of IFNAR1 ubiquitination as an effective strategy against acute local and generalized inflammatory syndromes.

## Discussion

Data presented here indicate that stimulated ubiquitination and degradation of IFNAR1 in the bone marrow-derived cells recruited to the foci of inflammation plays a major protective role during inflammation. Striking systemic and local phenotypes observed in *Ifnar1*^*SA*^ mice under etiologically and pathogenetically diverse types of inflammatory syndromes illustrate two important points. First, the results suggest that accelerated ubiquitination and ensuing downregulation of IFNAR1 in response to numerous stimuli may (at least to some extent) functionally mimic the complete loss of this receptor via genetic ablation. Under specific physiologic and pathologic scenarios, this accelerated degradation may result in a phenotypic equivalency between wild type and IFNAR1 knockout animals. Such equivalency should not be over-interpreted as the lack of role of IFN in a given physiologic process or a pathologic condition.

Second, given that upregulation of *Irf7* seen in *Ifnar1*^*SA*^ mice can contribute to further increases in ligands production (Supplementary Figs 1, 2, 9 and 15), the inability to eliminate IFNAR1 may create a feed-forward signaling amplification mechanism. Importantly, the augmented IFN signaling as well as phenotypes observed in *Ifnar1*^*SA*^ mice are evident despite numerous negative regulators of IFN pathway that function downstream of receptor and include the expression of signaling and transcriptional inhibitors including tyrosine phosphatases, suppressors of cytokine signaling, Sprouty, Ubp43, protein inhibitors of STAT, etc. (Coccia *et al*, 2006; Zhang ' Zhang, 2011; Sharma *et al*, 2012). This fact suggests that ubiquitination-dependent downregulation of IFNAR1 represents a major and critically important mechanism that attenuates the sensitivity of cells and tissues to IFN and plays a key role in limiting IFN-mediated immunopathology.

The mechanisms by which elimination of IFNAR1 in cells within inflamed tissues aids to restrict tissue damage and switch to recovery are likely to be complex. Temporarily limiting the output of IFN signaling is expected to attenuate the direct detrimental effects of IFN on cell growth and viability and affect IFN-induced p38-dependent (Katsoulidis *et al*, 2005) and NF-κB-regulated (Du *et al*, 2007) inflammatory pathways known to contribute to expression of additional inflammatory cytokines (Karin, 2005). Furthermore, previously published studies showed that IFNAR1 expression is essential for efficient IL6 signaling (Mitani *et al*, 2001). The produced cytokines/chemokines either directly kill cells or contribute to subsequent waves of recruitment of immune cells that are capable of further damaging the inflamed tissue. Pancreatic inflammation in mice deficient in IFNAR1 ubiquitination was paralleled with an altered balance between pro-inflammatory and anti-inflammatory cytokines (e.g. TNFα and IL10) and increased expression of CCL2 (Fig 3). Given that IFN directly induces transcription of *CCL2* (Buttmann *et al*, 2007) that mediates recruitment of inflammatory monocytes, which in turn produce TNFα and other inflammatory cytokines (Guha ' Mackman, 2001), it is plausible that down-regulation of IFNAR1 may help to restrict this pathway. Consistent with this possibility, a greater number of inflammatory monocytes/macrophages was observed in tissues from *Ifnar1*^*SA*^ mice (Fig 3 and Supplementary Fig 11).

In addition, a dramatic deficiency in tissue repair and regeneration seen in chronic pancreatitis model (Fig 4 and Supplementary Fig 12) suggests that other mechanisms affecting mesenchymal stem cells or/and endothelial progenitors might be involved as well. In light of demonstrated resistance of endothelial cells from *Ifnar1*^*SA*^ mice to VEGF-induced angiogenesis (Zheng *et al*, 2011b) along with observations that elevated levels of VEGF are protective against tissue injury in human patients with acute pancreatitis (Ueda *et al*, 2006), the role of efficient IFNAR1 ubiquitination in ensuring an adequate blood supply to the regenerating tissues might be important. Future studies delineating the mechanisms by which elimination of IFNAR1 aids to tissue recovery are warranted.

The practical relevance of the mechanisms utilizing IFNAR1 elimination to attenuate the responsiveness of tissues to IFN in hepatitis is underscored by several clinical observations. Despite the use of IFN in treatment of chronic viral hepatitis caused by hepatitis C and B viruses, the hepatic side effects including exacerbation of liver damage after IFN administration has been widely reported (Dusheiko, 1997). We proposed previously that the interventions aimed to enable IFNAR1 to withstand inflammatory elimination may increase the efficacy of IFN against tumors (Huangfu *et al*, 2012). Our current results argue to expand the clinical considerations for preventive stimulation of IFNAR1 turnover through PKD2/p38 induction in order to further limit the harmful effects of IFN.

Indeed, treatment with LPA capable of inducing IFNAR1 ubiquitination protected wild type but not *Ifnar1*^*SA*^ animals from toxic hepatitis and fulminant generalized inflammation (Fig 7). These data provide a proof of principle for pharmacologic downregulation of IFNAR1 to be used against acute life-threatening inflammatory conditions where the concerns for attenuated anti-viral or anti-tumor defenses are secondary. Similar to LPA, numerous natural and synthetic agonists of G-protein coupled receptors are capable of activating PKD2 and p38 kinases (Rosenbaum *et al*, 2009; Tilley, 2011). Some of these agents and their receptors were already shown to play a role in regulating the severity of inflammation (Ohta ' Sitkovsky, 2001). The usefulness of these receptors' agonists may be helped by the fact that these receptors are expressed either ubiquitously or in a tissue-specific manner (Jacoby *et al*, 2006) thereby affording systemic or selective protective effects.

Studies described here uncovered an endogenous mechanism by which inflammatory process confines its harmful phase and enables the transition to tissue restoration through promoting IFNAR1 ubiquitination and degradation. In addition, mimicking this natural mechanism by preemptive induction of p38 and PKD2 kinases that leads to stimulation of IFNAR1 ubiquitination elicits a tissue protective effect. Pharmacologic stimulation of IFNAR1 ubiquitination and degradation represents a fundamentally novel strategy to limit the extent of tissue damage and accelerate healing and restoration of the function. Given that modulators of the G-protein coupled receptors comprise the most populous class of currently available drugs (Rosenbaum *et al*, 2009; Wang ' Lewis, 2013), the validation of this concept and practical applications to eliminate IFNAR1 in acute life-threatening inflammatory syndromes is likely to be imminent.

## Materials and Methods

### Mice and inflammation modeling

The Institutional Animal Care and Use Committee (IACUC) of the University of Pennsylvania approved all animal procedures of disease modeling, blood collection, euthanasia and tissue harvesting (protocols #802558, 803995, 802868, 803699, and 804209). Eight to 10 weeks old female littermate mice including wild type (*Ifnar1*^*+/+*^) mice, *Ifnar1*^*SA*^ (*Ifnar1*^*tm1.1Syfu*^) mice (Zheng *et al*, 2011b) and *Ifnar1*^*−/−*^ mice (Roth-Cross *et al*, 2008; a generous gift of S. Weiss) were used in all experiments. We have previously described the generation of *Ifnar1*^*SA*^ knock-in mice (Zheng *et al*, 2011b) from the C57Bl/6 ES cells previously targeted with the knock-in vector [described in (Liu *et al*, 2009b)] and then treated with Cre to remove the neo marker *in vitro*. Whereas cells from heterozygous mice (*Ifnar1*^*+/SA*^) were used in previous publications (Qian *et al*, 2011; Zheng *et al*, 2011b), all of our current experiments were carried out using animals/cells that had both wild type alleles replaced by homozygous *Ifnar1*^*SA*^.

All mice were on 100% C57Bl/6 background (*Ifnar1*^*SA*^ were generated in C57Bl/6 embryonic stem cells (Liu *et al*, 2009b) and maintained in this background; *Ifnar1*^*−/−*^ mice were back-crossed into C57Bl/6 background for more than 20 generations) and were homozygous for indicated *Ifnar1* alleles. For generalized inflammation model, mice were treated with LPS (10.5 mg/kg, intraperitoneal injection) and sacrificed when they became moribund and displayed the loss of righting reflex, loss of >20% of body weight and non-responsiveness to footpad compression as previously described (Karaghiosoff *et al*, 2003). Bone marrow harvesting and transfers were carried out as described elsewhere (Pear *et al*, 1998). Blood cell and plasma chemistry analyses (including AST/ALT assays) were carried out by at the Clinical Pathology Laboratory at the University of Pennsylvania Ryan Veterinary Hospital.

### Experimental pancreatitis

was induced as previously described (Reichert ' Rustgi, 2011; Reichert *et al*, 2013). Briefly, acute pancreatitis was induced in by 8 hourly intraperitoneal injections of caerulein (50 μg/kg) for two consecutive days. Blood was collected from the tail vein at 3 or 7 days after the first injection. At this point, mice were sacrificed and pancreata were harvested and processed for mRNA isolation and either frozen section-immunohistochemistry or formalin-fixation and subsequent hematoxylin-eosin staining. Amylase assays were carried out as per manufacturer instructions (QuantiChrom α-Amylase Assay Kit). Chronic pancreatitis was induced by injections of caerulein (50 μg/kg per day for 5 days a week for 4 weeks). At the end of every week (day 5) blood was collected from the tail vein. Mice were sacrificed after 4 weeks and pancreata were harvested and analyzed as indicated above.

### Experimental hepatitis

To induce autoimmune hepatitis, mice were injected intravenously with Concanavalin A (20 mg/kg) or vehicle (sterile saline) and sacrificed 10 h later for blood and tissues harvesting. To induce toxic hepatitis, mice were injected intraperitoneally with CCl_4_ (0.5 ml/kg) or vehicle (olive oil) and sacrificed after 48 h. Alternatively, mice were injected with acetaminophen (APAP, 150 mg/kg, i.p.) and sacrificed after 24 h. Blood was processed for ALT/AST analyses. Livers were either flash frozen for extracting mRNA or fixed in 4% formalin for hematoxylin and eosin staining. Histopathologic analyses were carried in a double blinded manner by a qualified veterinary pathologist (A.B.). Quantification of the lesions was carried out by counting the number of lesions per field; an average of ten fields per mouse and five mice per group were scored. The assessment of the severity of hepatitis was done by evaluating tissues on a scale of 0–3 where the scores were representative of grades of 0 (none/minimal inflammation), 1 (mild hepatitis), 2 (moderate hepatitis with limited necrotic foci) and 3 (severe hepatitis that includes marked necrosis and displays multifocal to coalescing hepatocellular hyperplasia and hypertrophy with vacuolation).

### Statistical analysis

All data are expressed as the mean ± s.d. (standard deviation). Number of samples is shown in figures or/and figure legends for each experimental cohort. Statistical analyses were performed using Microsoft Excel or GraphPad Prism software, and differences were determined by an unpaired two-tailed Student's *t*-test. A *P*-value< 0.05 was considered statistically significant.

**The paper explained**

**Problem**

Inflammation is an adaptive process that combines the counteractive elements of tissue destruction versus healing to limit and eventually eliminate the harmful effects of irritants and infectious agents. Initial stages of inflammation that exacerbate tissue damage are often followed by subsequent regeneration/repair of tissue to restore its function. While failure to progress to these later stages contributes to development of many human diseases, the mechanisms limiting tissue injury and promoting the transition to the restorative phase are poorly understood. Type 1 interferons (IFN) protect the organism from viruses and tumors yet elicit tissue toxicities often being manifested in patients with tumors or chronic viral infections treated with IFN-based drugs. All effects of IFN are mediated by its cell surface receptor, whose levels are regulated by phosphorylation-dependent ubiquitination and degradation of the IFNAR1 chain. Importantly, these processes depriving cells from ability to respond to their future encounter with IFN can be stimulated by inflammatory stimuli *in vitro*. Should this regulation occur *in vivo*, it would pose a medical problem of reduced efficacy of pharmaceutical IFN in inflamed tissues. Furthermore, the very existence of such mechanism is counterintuitive for the host defense as it may limit the anti-viral and anti-tumorigenic benefits of endogenously produced IFN within the regions of inflammation. Hence, we sought to investigate the manifestation and biological significance of inflammation-induced IFNAR1 ubiquitination and downregulation *in vivo*.

**Results**

Induction of pancreatitis, hepatitis or generalized inflammation robustly downregulated IFNAR1 in mice. This downregulation was not seen in the knock-in mice incapable of stimulating IFNAR1 ubiquitination. Importantly, these mice were highly susceptible to acute and chronic pancreatitis, toxic and autoimmune hepatitis and bacterial endotoxin-induced septic shock. Altered balance of pro-inflammatory/anti-inflammatory cytokines and specific chemokines resulting in the recruitment of inflammatory monocytes was observed in pancreatic tissues of IFNAR1 ubiquitination-deficient mice. These mice displayed extensive damage and persistent immune infiltration of inflamed tissues, and, in the case of generalized inflammation, overwhelming mortality. Under conditions of chronic pancreatitis, these mice also exhibited a dramatically impaired ability to repair the pancreatic tissue and restore its anatomical and functional integrity. Experiments using bone marrow transplantation determined that inability to ubiquitinate and degrade IFNAR1 in hematopoietic compartment is responsible for excessive pancreatic and hepatic inflammatory phenotypes. Importantly, pharmacologic stimulation of protein kinases that stimulate IFNAR1 ubiquitination was protective against from toxic hepatitis and fulminant generalized inflammation in wild type but not in IFNAR1 ubiquitination-deficient mice.

**Impact**

Studies described here uncovered an endogenous mechanism by which inflammatory process confines its harmful phase and enables the transition to tissue restoration through promoting IFNAR1 ubiquitination and degradation. In addition, mimicking this natural mechanism by preemptive induction of protein kinases that leads to stimulation of IFNAR1 ubiquitination elicits a tissue protective effect. Pharmacologic stimulation of IFNAR1 ubiquitination and degradation represents a fundamentally novel strategy to limit the extent of tissue damage and accelerate healing and restoration of the function. Furthermore, a similar strategy could be potentially used to temper the toxic side effects of the IFN-based drugs. Given that many of the currently available drugs are potentially capable of stimulating IFNAR1 ubiquitination, the validation of this concept and practical applications to eliminate IFNAR1 in acute life-threatening inflammatory syndromes is likely to be imminent.

## For more information

http://www.genecards.org/cgi-bin/carddisp.pl?gene=IFNAR1

http://www.ncbi.nlm.nih.gov/gene/3454

http://www.ncbi.nlm.nih.gov/gene/15975

http://www.med.upenn.edu/apps/faculty/index.php/g20001863/p2152997
