# Supplementary material for: Triggering ubiquitination of IFNAR1 protects tissues from inflammatory injury
Source: EMBO Mol Med. 2014 Jan 31;6(3):384–97. doi: 10.1002/emmm.201303236 (PMC3958312; doi:10.1002/emmm.201303236)
Supplement: Supplementary file 1 [file emmm0006-0384-sd1.pdf]

Triggering ubiquitination of IFNAR1 protects tissues from inflammatory injury

Sabyasachi Bhattacharya, Kanstantsin V. Katlinski, Maximilian Reichert, Shigetsugu Takano, Angela Brice, Bin Zhao, Qiujing Yu, Hui Zheng, Christopher J. Carbone, Yuliya V. Katlinskaya, N. Adrian Leu, Kelly A. McCorkell, Satish Srinivasan, Melanie Girondo, Hallgeir Rui, Michael J. May, Narayan G. Avadhani, Anil K. Rustgi, and Serge Y. Fuchs

Corresponding author: Serge Fuchs, University of Pennsylvania

Review timeline:

Submission date:	26 June 2013
Accepted:	13 December 2013

Editor: Céline Carret

Transaction Report:

No Peer Review Process File is available with this article, as the authors have chosen not to make the review process public in this case.
