# Supplementary material for: Triggering ubiquitination of IFNAR1 protects tissues from inflammatory injury
Source: EMBO Mol Med. 2014 Jan 31;6(3):384–97. doi: 10.1002/emmm.201303236 (PMC3958312; doi:10.1002/emmm.201303236)

Source data: Figure 1 D

Panel 1 (Ubi)

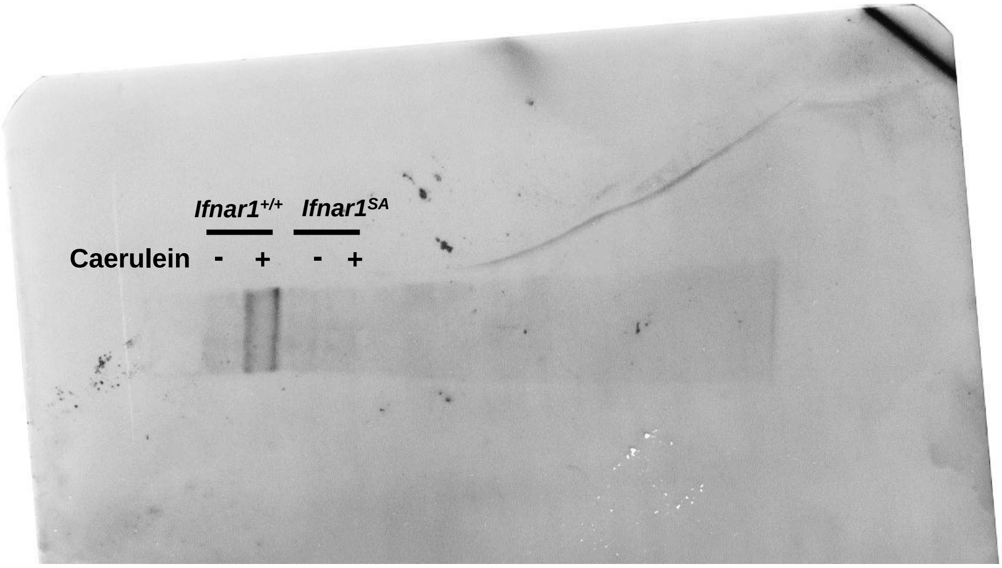

Panel 2 (IFNAR1)

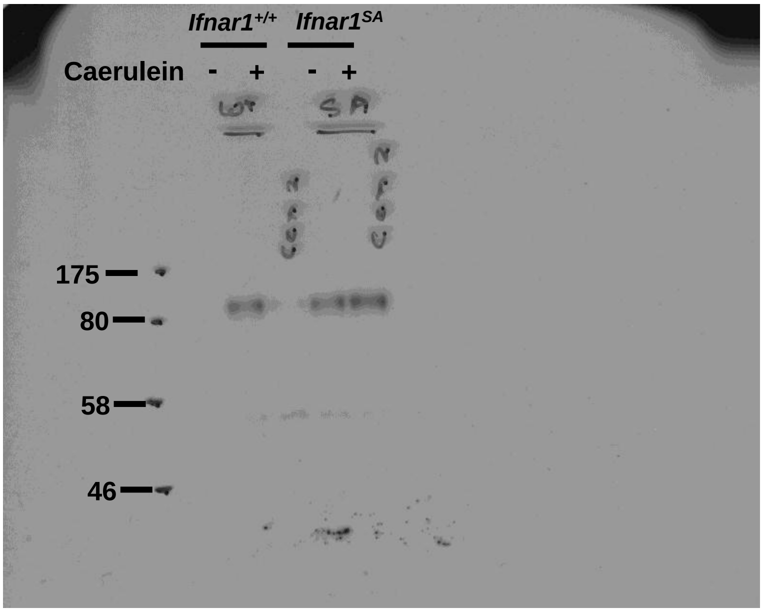

Source data: Figure 1 D

Panel 3 (p-p38)

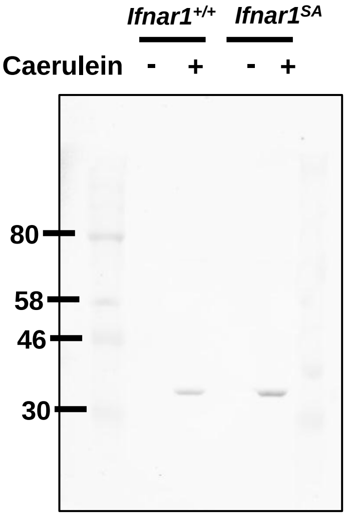

Panel 4 (p38)

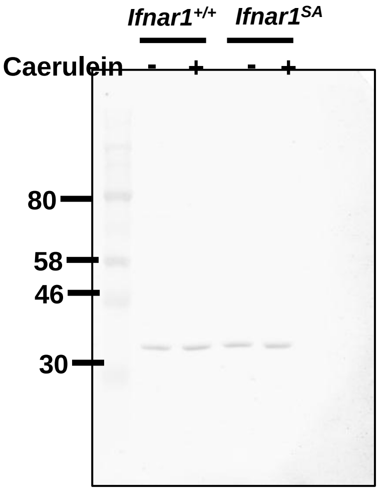

Source data: Figure 1 D

Panel 5 (STAT1)

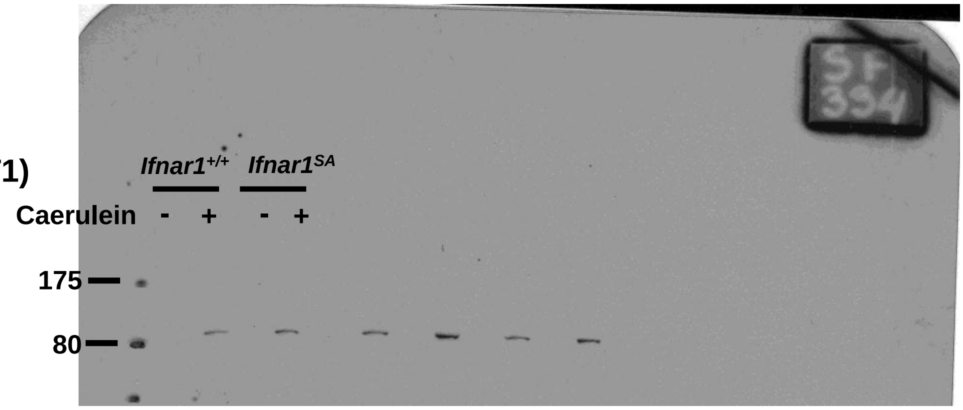

Panel 6 (PKR)

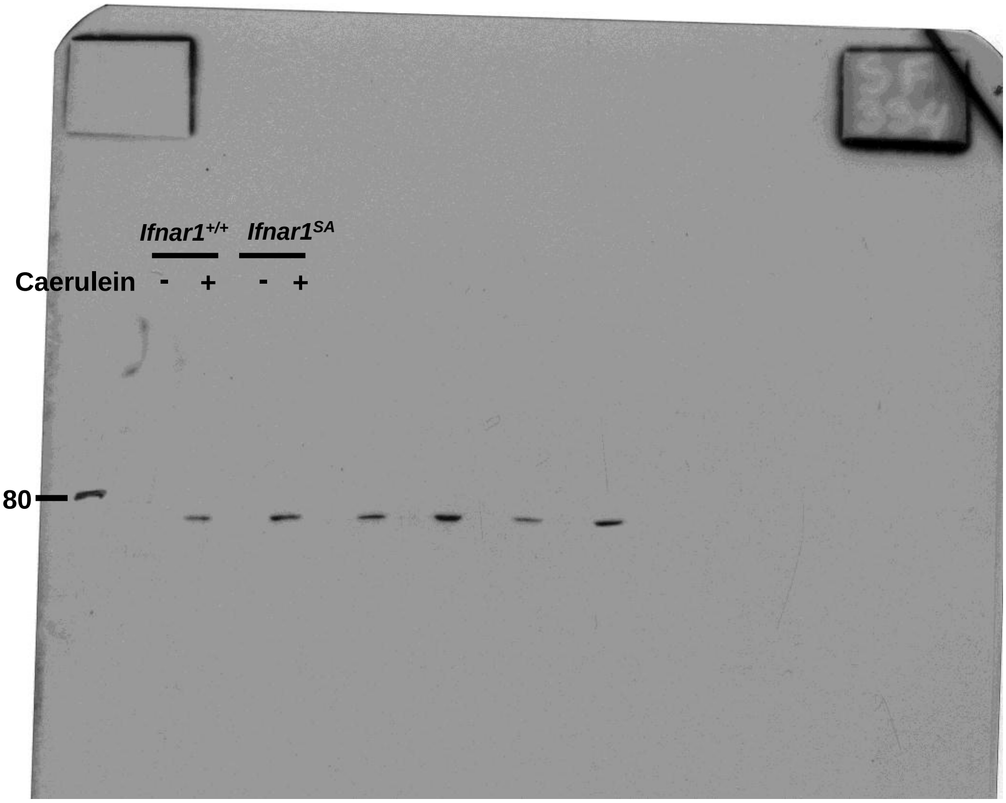

Supplement: Supplementary file 2 [file emmm0006-0384-sd2.pdf]
