# Supplementary figures and images for: Triggering ubiquitination of IFNAR1 protects tissues from inflammatory injury
Source: EMBO Mol Med. 2014 Jan 31;6(3):384–97. doi: 10.1002/emmm.201303236 (PMC3958312; doi:10.1002/emmm.201303236)

Panel used in the  
revised figure

Ifnar1<sup>+/+</sup>  
w. Concavalin

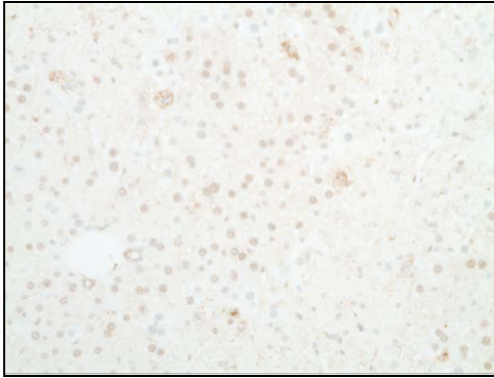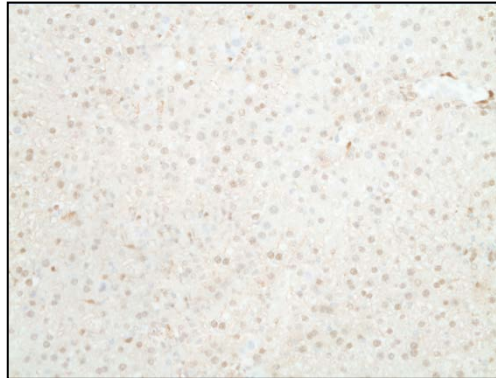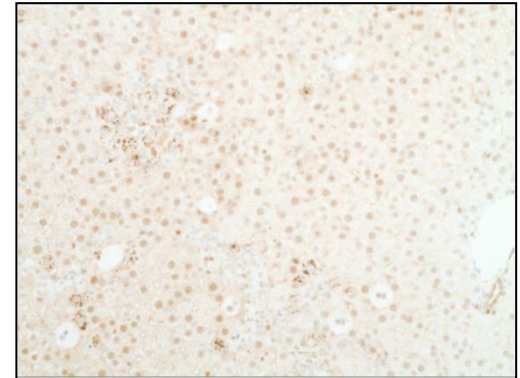

Ifnar1<sup>SA</sup>  
w. Saline

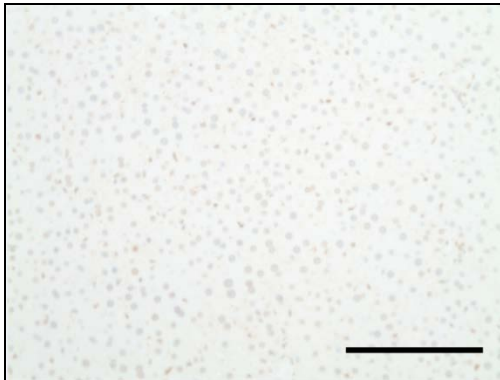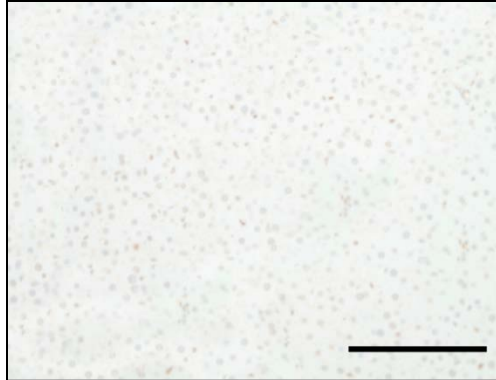

Supplement: Supplementary file 3 [file emmm0006-0384-sd3.pdf]
