# Supplementary material for: Triggering ubiquitination of IFNAR1 protects tissues from inflammatory injury
Source: EMBO Mol Med. 2014 Jan 31;6(3):384–97. doi: 10.1002/emmm.201303236 (PMC3958312; doi:10.1002/emmm.201303236)

Source data: Figure 7A

Panel 1 Ub

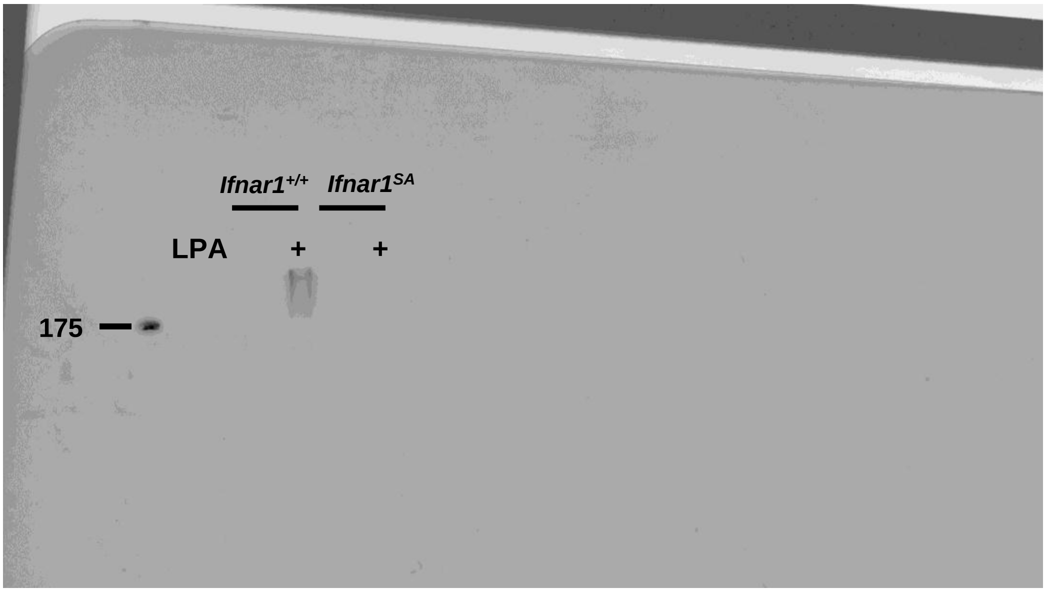

Panel 2 p-IFNAR1

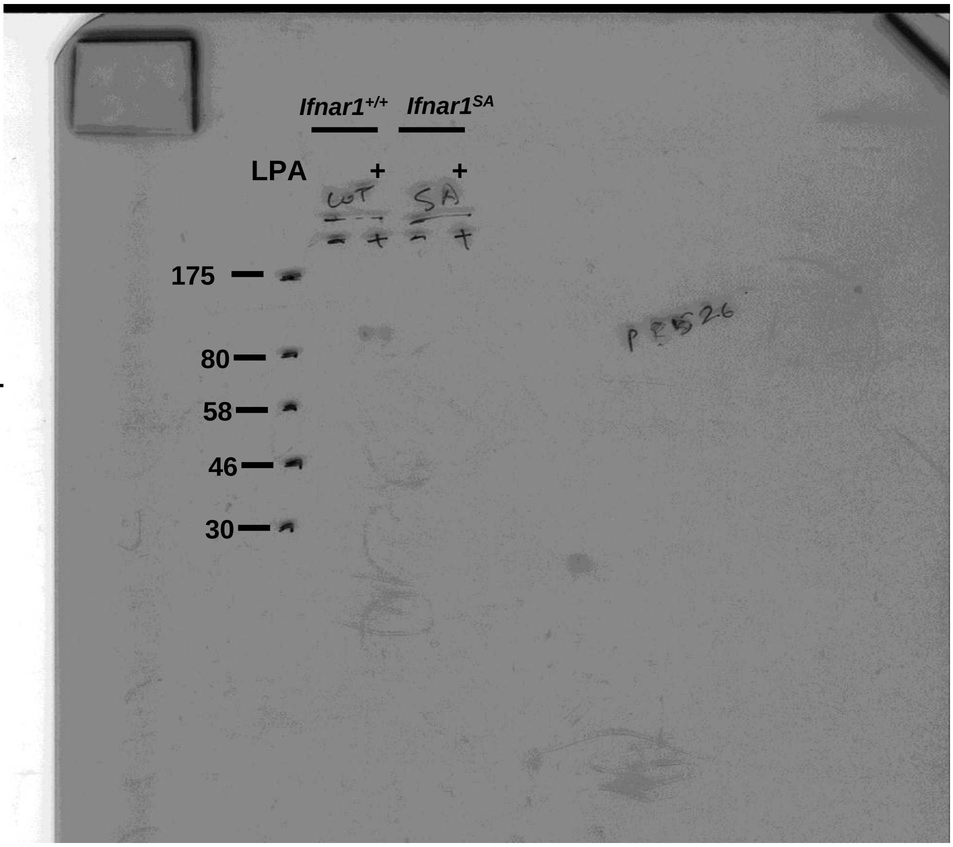

Source data: Figure 7A

Panel 3 IFNAR1

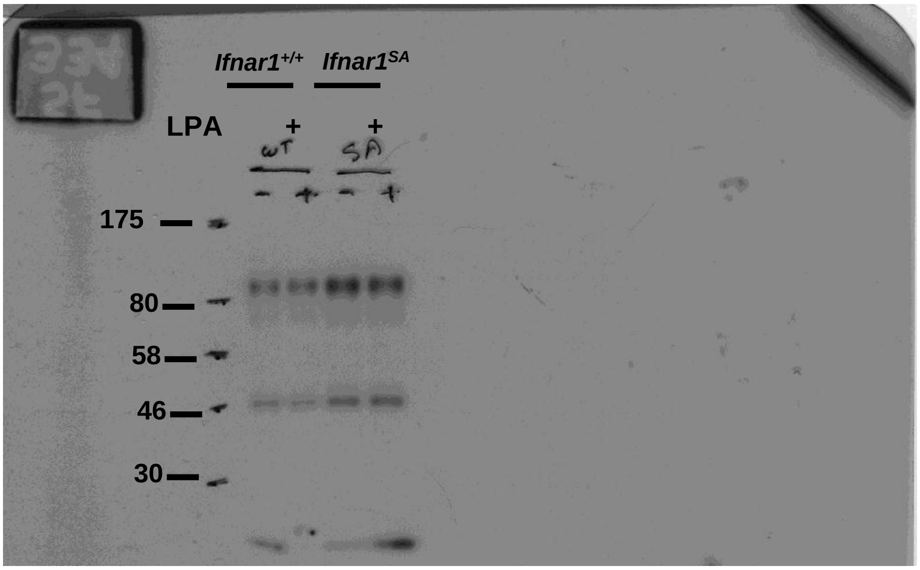

Source data: Figure 7A

Panel 4 and 6
pPKD2 and p-p38

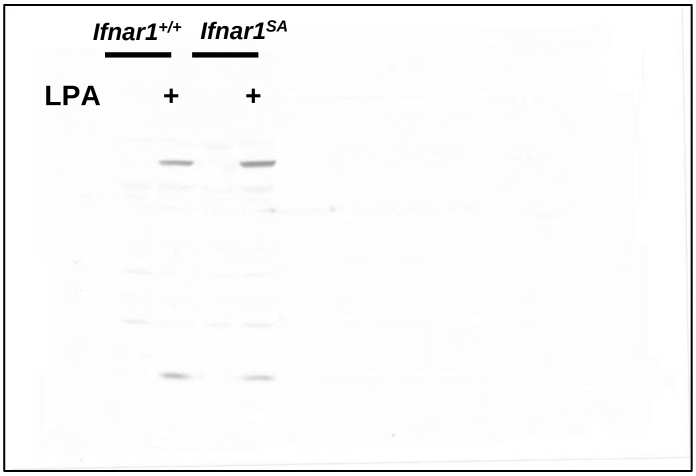

Panel 5 and 7
PKD2 and p38

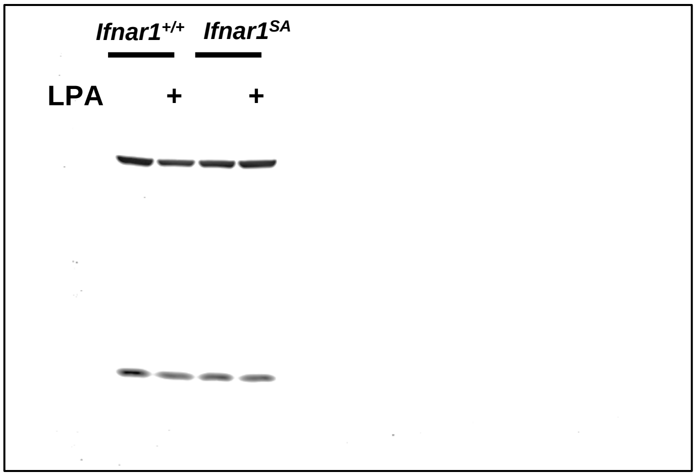

Source data: Figure 7C

Panel 1 p-STAT1

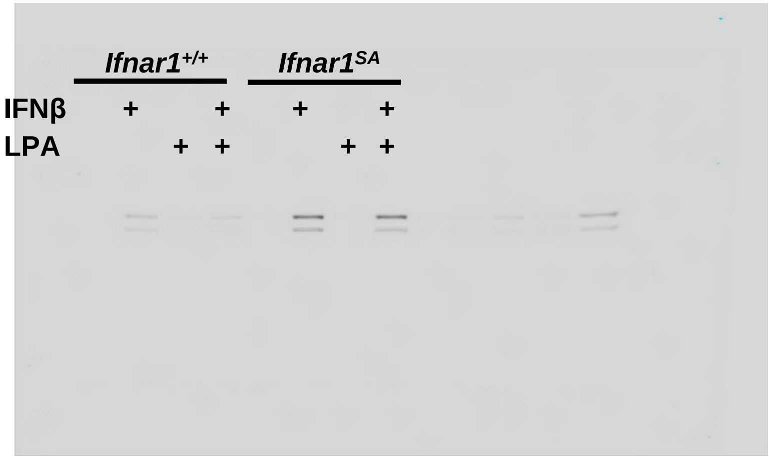

Panel 2 STAT1

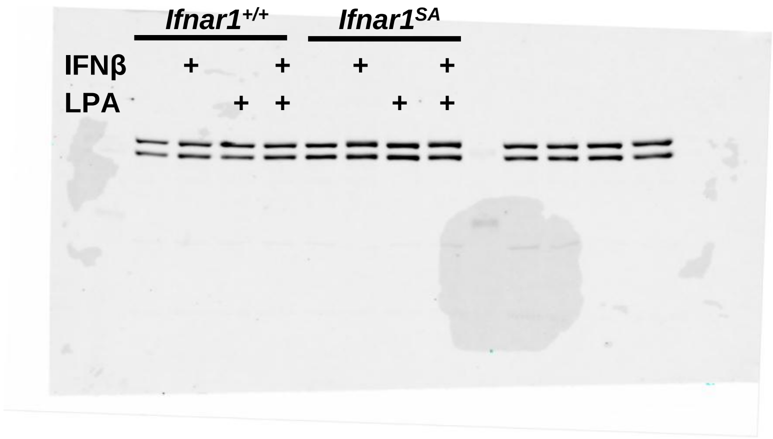

Supplement: Supplementary file 4 [file emmm0006-0384-sd4.pdf]
