# Supplementary material for: Triggering ubiquitination of IFNAR1 protects tissues from inflammatory injury
Source: EMBO Mol Med. 2014 Jan 31;6(3):384–97. doi: 10.1002/emmm.201303236 (PMC3958312; doi:10.1002/emmm.201303236)
Supplement: Supplementary file 5 [file emmm0006-0384-sd5.pdf]

S1

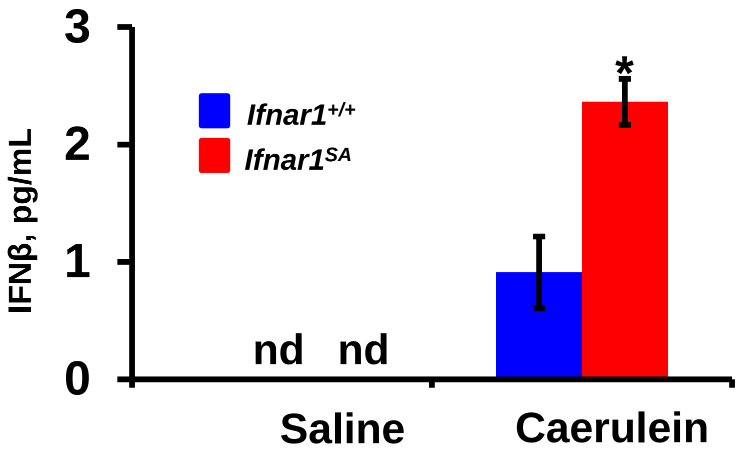

Figure S1: ELISA analyses of the levels of IFNβ (in pg/mL) in blood plasma of indicated mice (n=3 for each genotype) three days post injection with saline or caerulein. * - $p<0.001$ compared to saline treatments. Nd, not detectable (below 0.94 pg/mL).
