# Supplementary material for: Triggering ubiquitination of IFNAR1 protects tissues from inflammatory injury
Source: EMBO Mol Med. 2014 Jan 31;6(3):384–97. doi: 10.1002/emmm.201303236 (PMC3958312; doi:10.1002/emmm.201303236)
Supplement: Supplementary file 6 [file emmm0006-0384-sd6.pdf]

S2

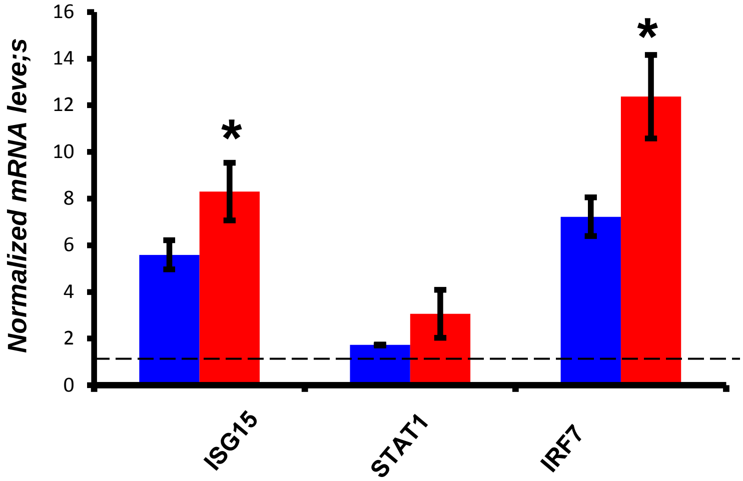

Figure S2: Fold induction of mRNA of *Isg15, Stat1, and Irf7* (normalized per *β-actin* mRNA) in pancreata from indicated mice (n=3 for each genotype) harvested 3 days after caerulein injections. Normalized mRNA levels in mice that received saline were assigned a value of 1.0 (dashed horizontal line). Asterisk: $p<0.05$ in comparison with wild type mice.
