# Supplementary material for: Triggering ubiquitination of IFNAR1 protects tissues from inflammatory injury
Source: EMBO Mol Med. 2014 Jan 31;6(3):384–97. doi: 10.1002/emmm.201303236 (PMC3958312; doi:10.1002/emmm.201303236)
Supplement: Supplementary file 8 [file emmm0006-0384-sd8.pdf]

S4

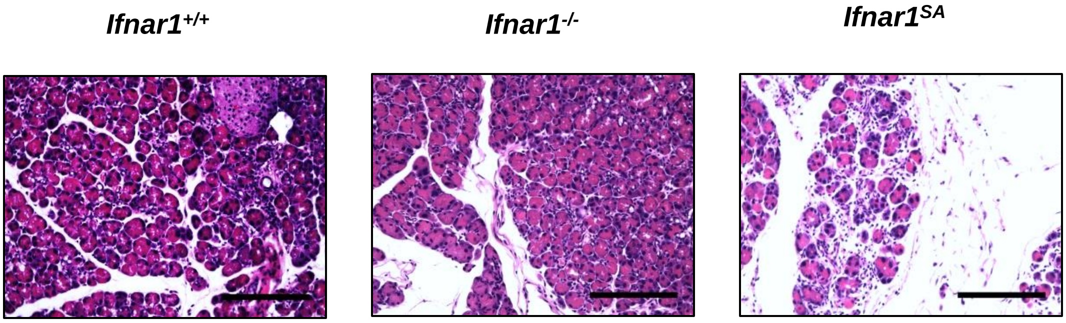

Figure S4: H&E staining of pancreata from mice of indicated genotypes at 3 days following caerulein injections.
