# Supplementary material for: Triggering ubiquitination of IFNAR1 protects tissues from inflammatory injury
Source: EMBO Mol Med. 2014 Jan 31;6(3):384–97. doi: 10.1002/emmm.201303236 (PMC3958312; doi:10.1002/emmm.201303236)
Supplement: Supplementary file 9 [file emmm0006-0384-sd9.pdf]

S5

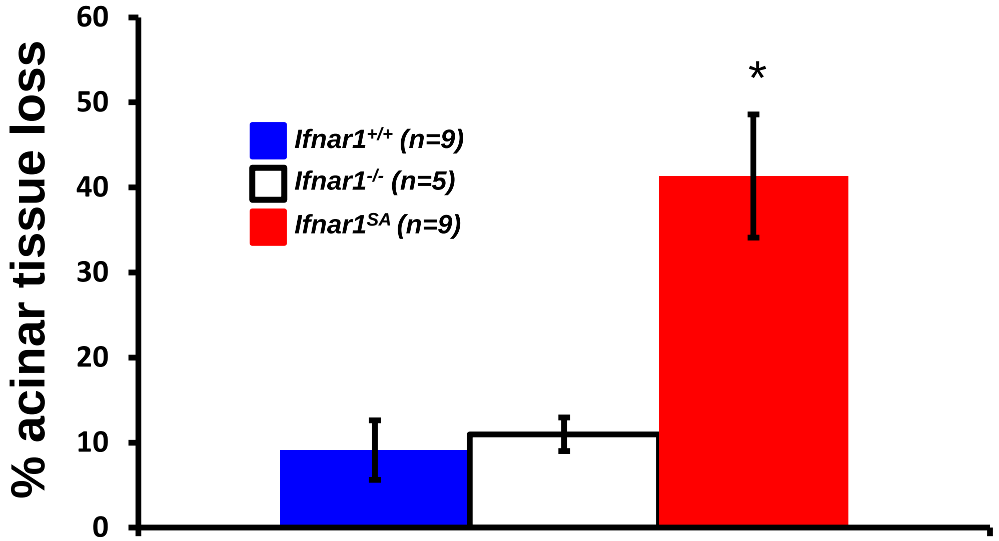

Figure S5: Quantification of acinar tissue damage (as % of loss of acinar tissue at three days after caerulein treatment compared to saline-treated animals). *: $p<0.05$
