# Supplementary material for: Triggering ubiquitination of IFNAR1 protects tissues from inflammatory injury
Source: EMBO Mol Med. 2014 Jan 31;6(3):384–97. doi: 10.1002/emmm.201303236 (PMC3958312; doi:10.1002/emmm.201303236)
Supplement: Supplementary file 10 [file emmm0006-0384-sd10.pdf]

S6

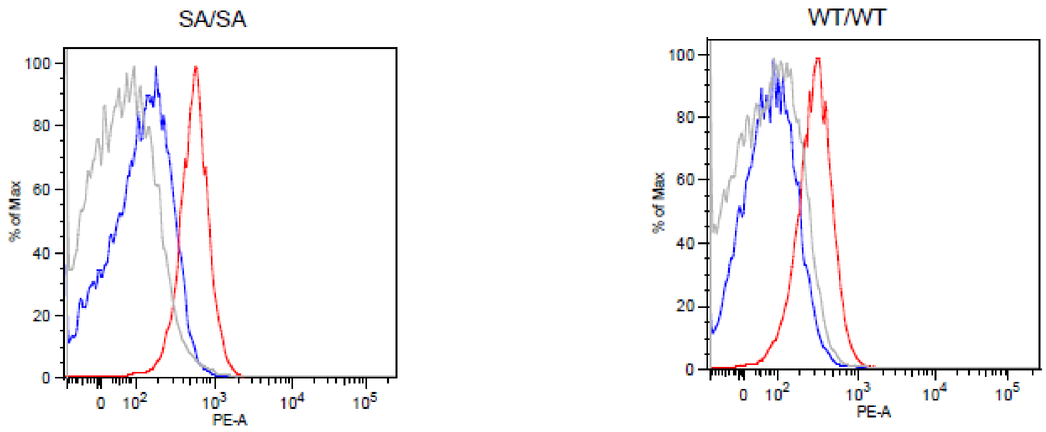

Label		Sample	Median:PE-A
ISO	(gray)	Specimen_001_Tube_001SA iso.fcs	57.5
Untreated	(red)	Specimen_001_Tube_003SAMED5.fcs	561
Trypsin 0.125% 5 min	(blue)	Specimen_001_Tube_002 SA TRYP5.fcs	129

Label		Sample	Median:PE-A
ISO	(gray)	Specimen_001_Tube_006WTISO.fcs	61.2
Untreated	(red)	Specimen_001_Tube_008WTMED5.fcs	302
Trypsin 0.125% 5 min	(blue)	Specimen_001_Tube_007WTTRYP5.fcs	73.4

Figure S6: FACS analysis of IFNAR1 cell surface levels in splenocytes from indicated mice subjected or not to treatment with trypsin (0.125% solution for 5 min, blue line). ISO: isotype antibody control (gray line).
