# Supplementary material for: Triggering ubiquitination of IFNAR1 protects tissues from inflammatory injury
Source: EMBO Mol Med. 2014 Jan 31;6(3):384–97. doi: 10.1002/emmm.201303236 (PMC3958312; doi:10.1002/emmm.201303236)
Supplement: Supplementary file 11 [file emmm0006-0384-sd11.pdf]

S7

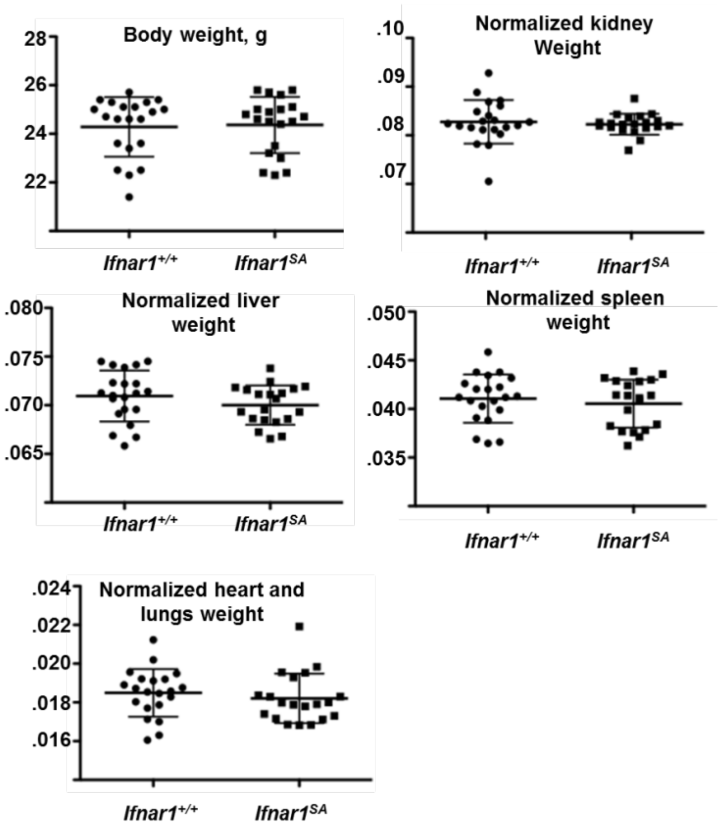

Figure S7: Body weight (g) and relative organ weight (normalized per body weight) of wild type and *Ifnar1*^{SA} mice.
