# Supplementary material for: Triggering ubiquitination of IFNAR1 protects tissues from inflammatory injury
Source: EMBO Mol Med. 2014 Jan 31;6(3):384–97. doi: 10.1002/emmm.201303236 (PMC3958312; doi:10.1002/emmm.201303236)
Supplement: Supplementary file 12 [file emmm0006-0384-sd12.pdf]

S8

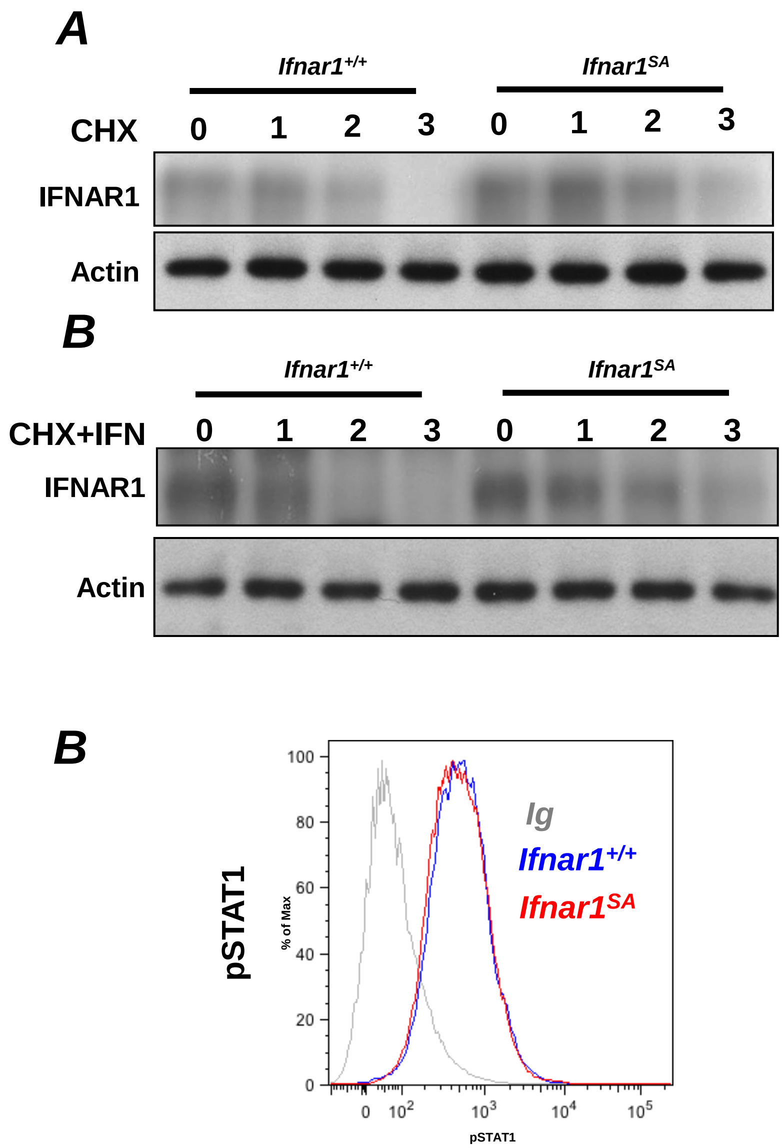

Figure S8: Characteristics of splenic cells from *Ifnar1*^{SA} mice. A. Degradation kinetics of IFNAR1 was analyzed by cycloheximide (CHX) chase in the upper panel. B. Lower panel depicts FACS analysis of intracellular STAT1 phosphorylation in the splenocytes from wild type or *Ifnar1*^{SA} mice. Ig, isotype antibody control.
