# Supplementary material for: Triggering ubiquitination of IFNAR1 protects tissues from inflammatory injury
Source: EMBO Mol Med. 2014 Jan 31;6(3):384–97. doi: 10.1002/emmm.201303236 (PMC3958312; doi:10.1002/emmm.201303236)
Supplement: Supplementary file 13 [file emmm0006-0384-sd13.pdf]

S9

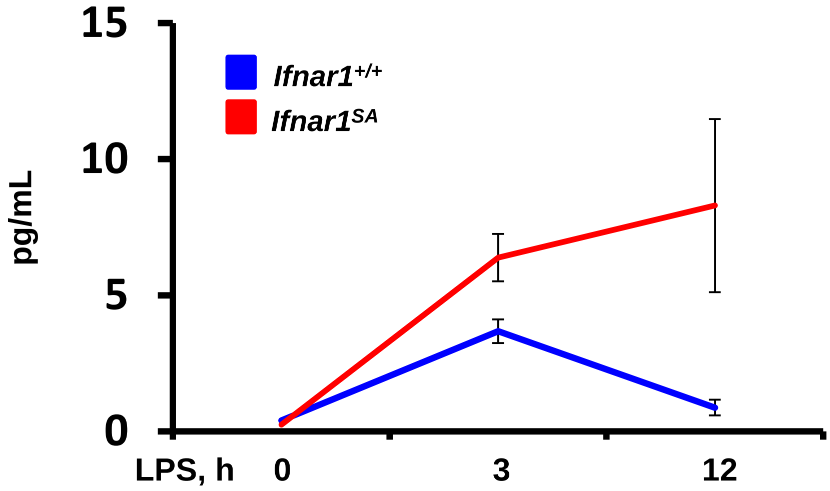

Figure S9: ELISA analyses of the levels of IFNβ (in pg/mL) in blood plasma of indicated mice (n=3 for each genotype) treated with saline (Sal) or LPS for 3 and 12h.
