# Supplementary material for: Triggering ubiquitination of IFNAR1 protects tissues from inflammatory injury
Source: EMBO Mol Med. 2014 Jan 31;6(3):384–97. doi: 10.1002/emmm.201303236 (PMC3958312; doi:10.1002/emmm.201303236)
Supplement: Supplementary file 14 [file emmm0006-0384-sd14.pdf]

S10

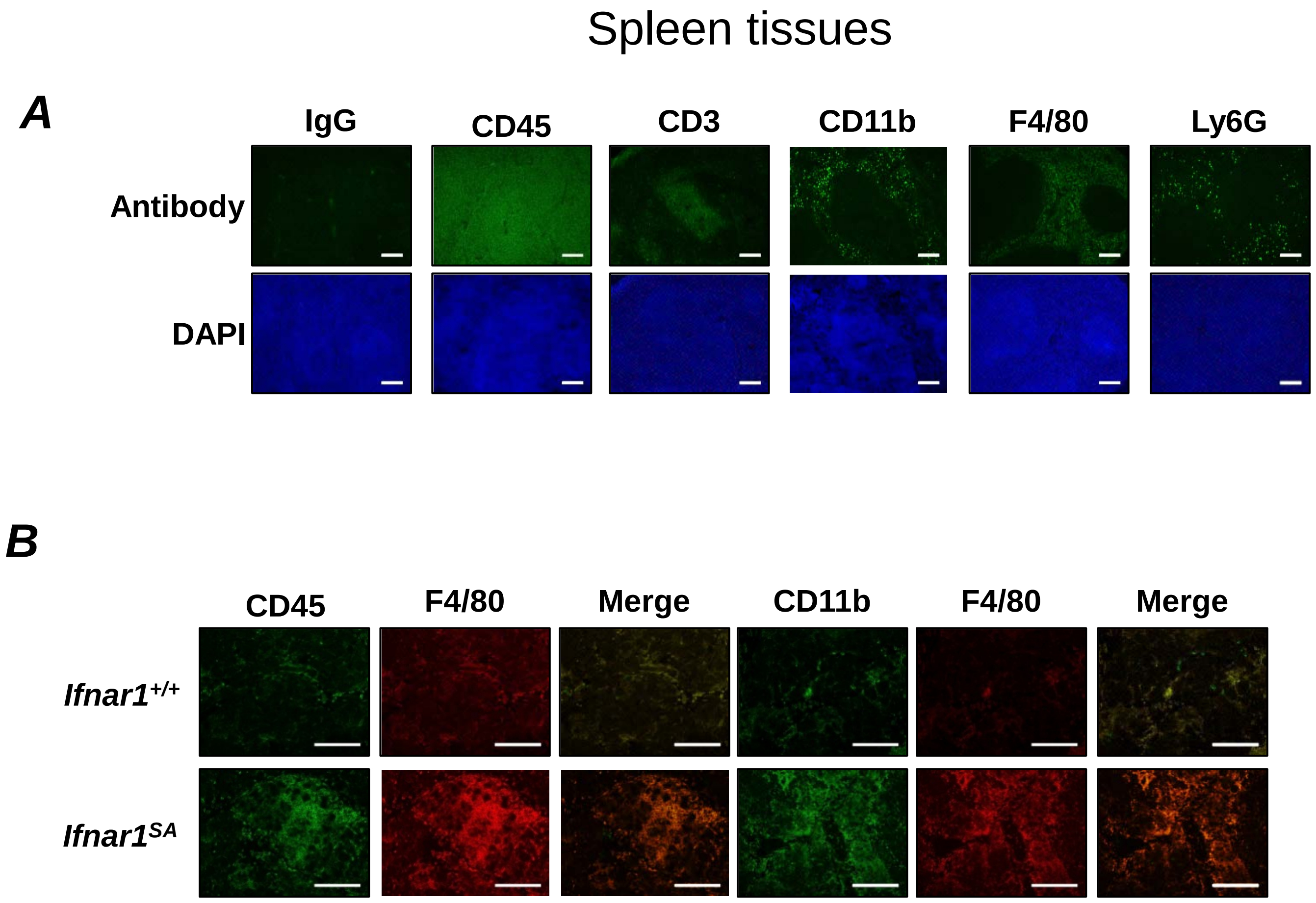

Figure S10: (A) Immunofluorescence analysis of adjacent serial cryosections from spleens from wild type mice using the indicated antibodies or control Ig. (B) Immunofluorescent analysis of areas of high leukocytic infiltration within indicated pancreata harvested 7 days post caerulein injection using indicated antibodies.
