# Supplementary material for: Triggering ubiquitination of IFNAR1 protects tissues from inflammatory injury
Source: EMBO Mol Med. 2014 Jan 31;6(3):384–97. doi: 10.1002/emmm.201303236 (PMC3958312; doi:10.1002/emmm.201303236)
Supplement: Supplementary file 15 [file emmm0006-0384-sd15.pdf]

S11

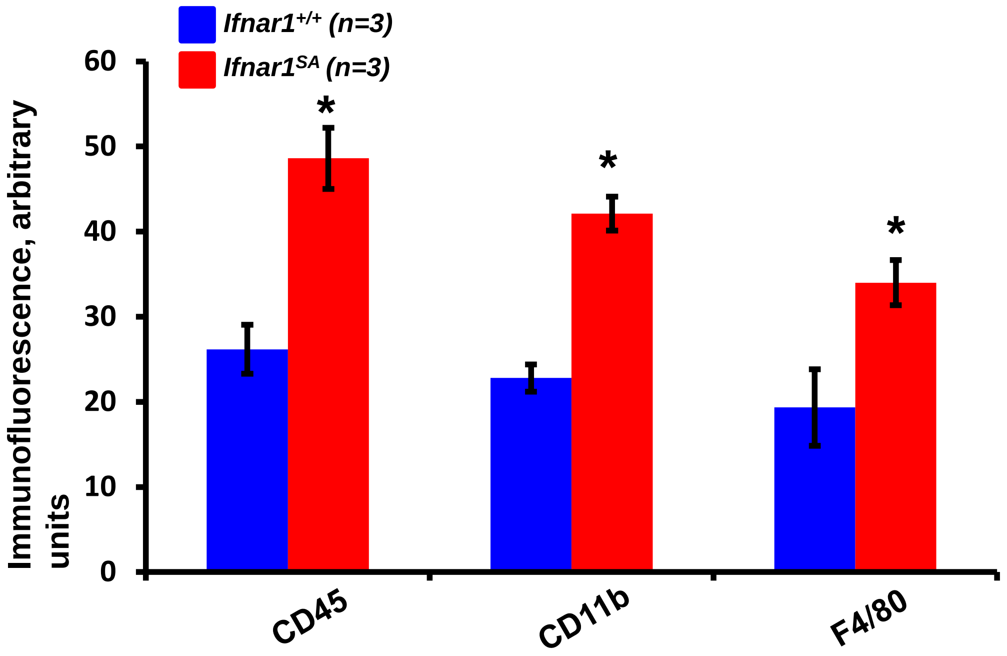

Figure S11: Relative intensity of immunofluorescence from indicated pancreatic tissues (in arbitrary units) analyzed by indicated antibodies was assessed using the ImageJ software. Data shown as average of nine images (three per mouse, n=3 mice) ± S.D. *: $p<0.05$
