# Supplementary material for: Triggering ubiquitination of IFNAR1 protects tissues from inflammatory injury
Source: EMBO Mol Med. 2014 Jan 31;6(3):384–97. doi: 10.1002/emmm.201303236 (PMC3958312; doi:10.1002/emmm.201303236)
Supplement: Supplementary file 16 [file emmm0006-0384-sd16.pdf]

S12

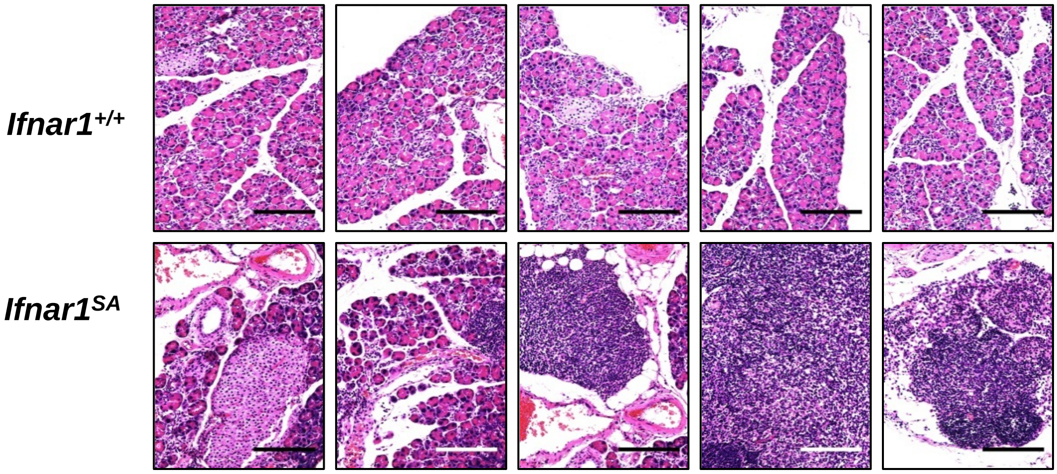

Figure S12: H&E staining of pancreata from mice of indicated genotypes (n=5 for each) at 4 weeks of continuous chronic caerulein treatment.
