# Supplementary material for: Triggering ubiquitination of IFNAR1 protects tissues from inflammatory injury
Source: EMBO Mol Med. 2014 Jan 31;6(3):384–97. doi: 10.1002/emmm.201303236 (PMC3958312; doi:10.1002/emmm.201303236)
Supplement: Supplementary file 17 [file emmm0006-0384-sd17.pdf]

S13

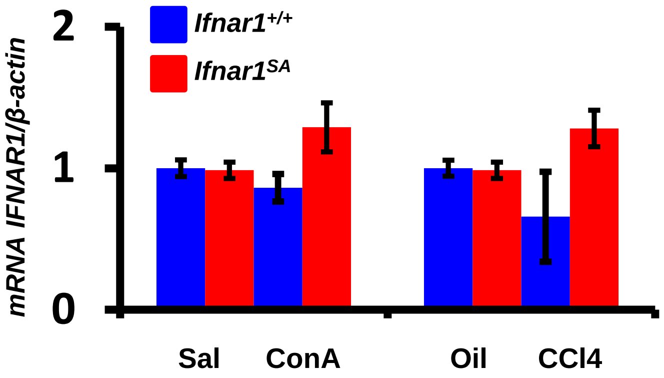

Figure S13: Fold induction of mRNA of *IFNAR1,* (normalized per *β-actin* mRNA) in liver from indicated mice (n=3 for each genotype). P>0.1 compared to vehicle treatments.
