# Supplementary material for: Triggering ubiquitination of IFNAR1 protects tissues from inflammatory injury
Source: EMBO Mol Med. 2014 Jan 31;6(3):384–97. doi: 10.1002/emmm.201303236 (PMC3958312; doi:10.1002/emmm.201303236)
Supplement: Supplementary file 18 [file emmm0006-0384-sd18.pdf]

S14

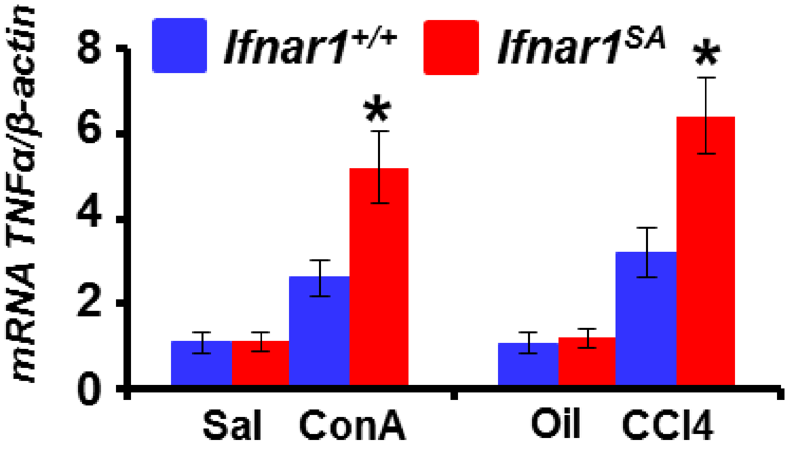

Figure S14: Relative levels of *Tnfα* mRNA normalized per *β-actin* mRNA in liver tissues from indicated mice (n=3 for each genotype).
