# Supplementary material for: Triggering ubiquitination of IFNAR1 protects tissues from inflammatory injury
Source: EMBO Mol Med. 2014 Jan 31;6(3):384–97. doi: 10.1002/emmm.201303236 (PMC3958312; doi:10.1002/emmm.201303236)
Supplement: Supplementary file 19 [file emmm0006-0384-sd19.pdf]

S15

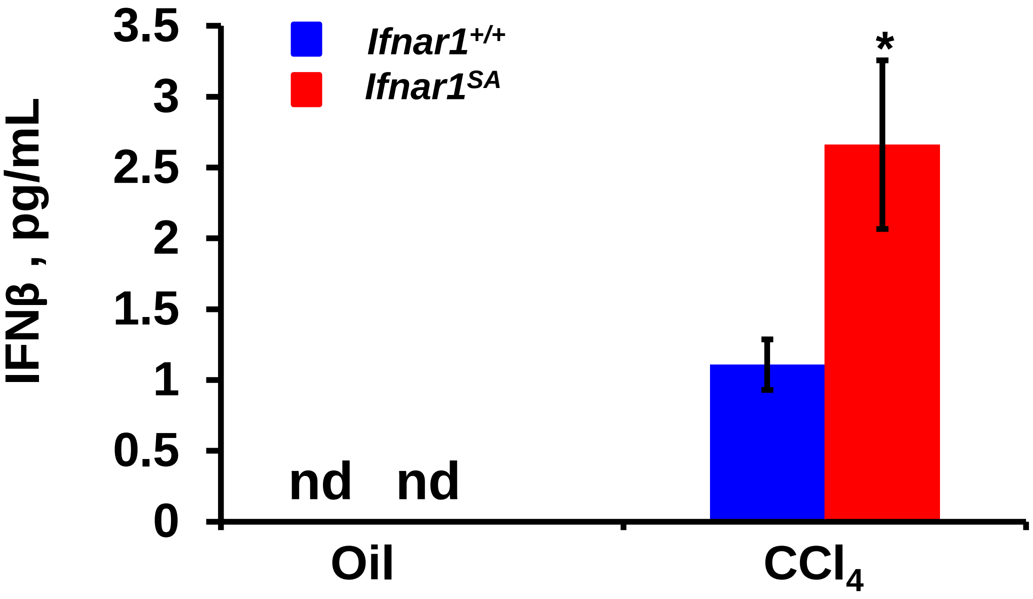

Figure S15: ELISA measurement of the levels of plasma IFNβ in indicated mice treated with CCl_4 or vehicle. *: $p<0.05$. nd, not detectable (below 0.94 pg/mL).
