# Supplementary material for: Triggering ubiquitination of IFNAR1 protects tissues from inflammatory injury
Source: EMBO Mol Med. 2014 Jan 31;6(3):384–97. doi: 10.1002/emmm.201303236 (PMC3958312; doi:10.1002/emmm.201303236)
Supplement: Supplementary file 20 [file emmm0006-0384-sd20.pdf]

S16

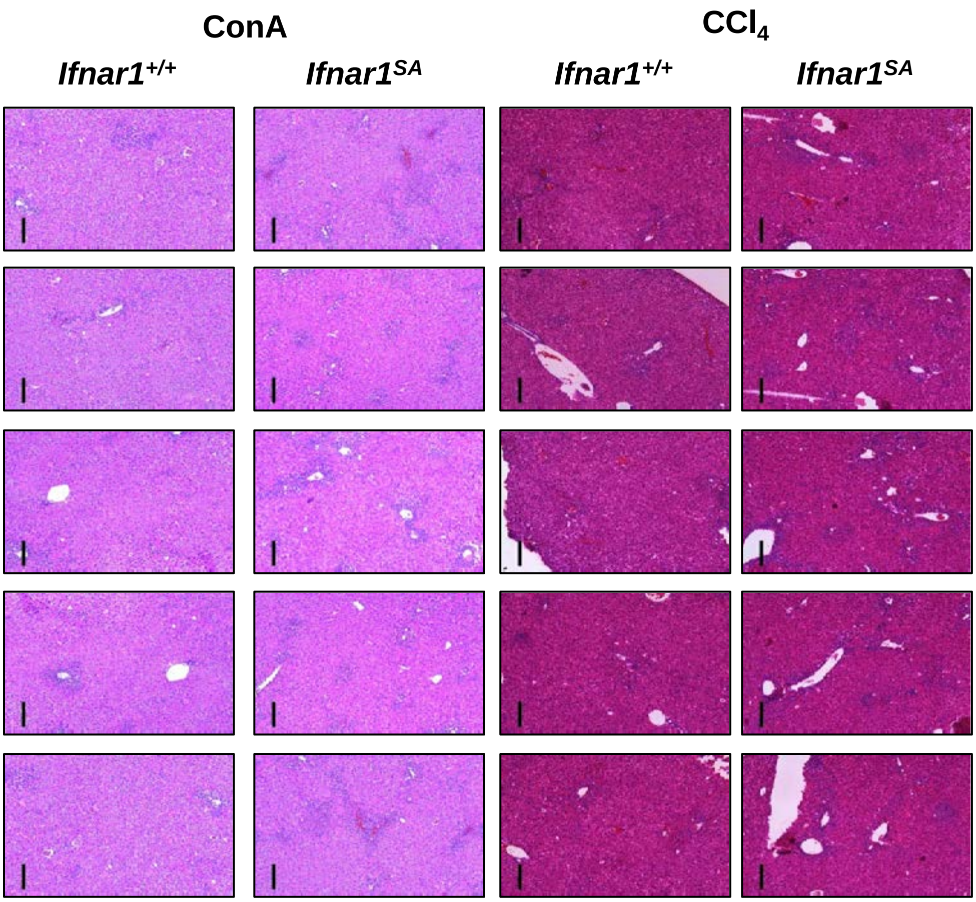

Figure S16: H&E staining of liver tissues from wild type or *Ifnar1*^{SA} mice (n=5 each) treated as indicated.
