# Supplementary material for: Triggering ubiquitination of IFNAR1 protects tissues from inflammatory injury
Source: EMBO Mol Med. 2014 Jan 31;6(3):384–97. doi: 10.1002/emmm.201303236 (PMC3958312; doi:10.1002/emmm.201303236)
Supplement: Supplementary file 21 [file emmm0006-0384-sd21.pdf]

S17

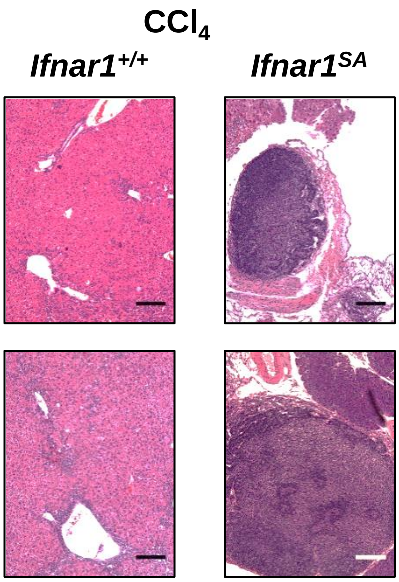

Figure S17: Areas of high density leukocyte infiltration observed on liver sections (H&E staining) of tissues from wild type or *Ifnar1*^{SA} mice treated with CCl_4.
