# Supplementary material for: Triggering ubiquitination of IFNAR1 protects tissues from inflammatory injury
Source: EMBO Mol Med. 2014 Jan 31;6(3):384–97. doi: 10.1002/emmm.201303236 (PMC3958312; doi:10.1002/emmm.201303236)
Supplement: Supplementary file 24 [file emmm0006-0384-sd24.pdf]

S20

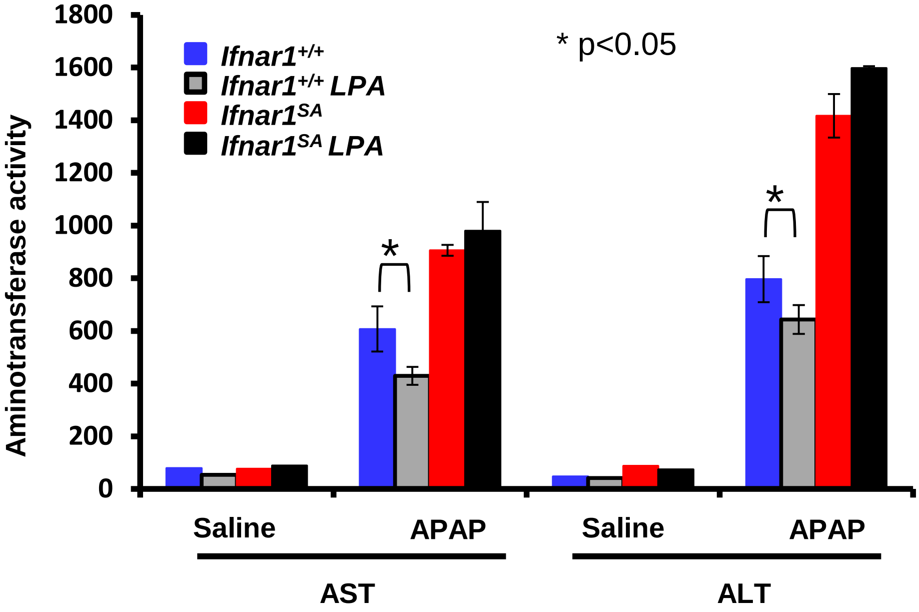

Figure S20: AST and ALT activity in plasma from *Ifnar1*$^{+}$ or *Ifnar1*^{SA} mice (n=4 each) collected 24 h after the injection of acetaminophen (APAP, 150mg/kg, i.p.). Where indicated, mice were also injected with LPA (10mg/kg, i.v.) at 8 and 16h after APAP treatment. *: p<0.05
