# Supplementary material for: Triggering ubiquitination of IFNAR1 protects tissues from inflammatory injury
Source: EMBO Mol Med. 2014 Jan 31;6(3):384–97. doi: 10.1002/emmm.201303236 (PMC3958312; doi:10.1002/emmm.201303236)
Supplement: Supplementary file 25 [file emmm0006-0384-sd25.pdf]

S21

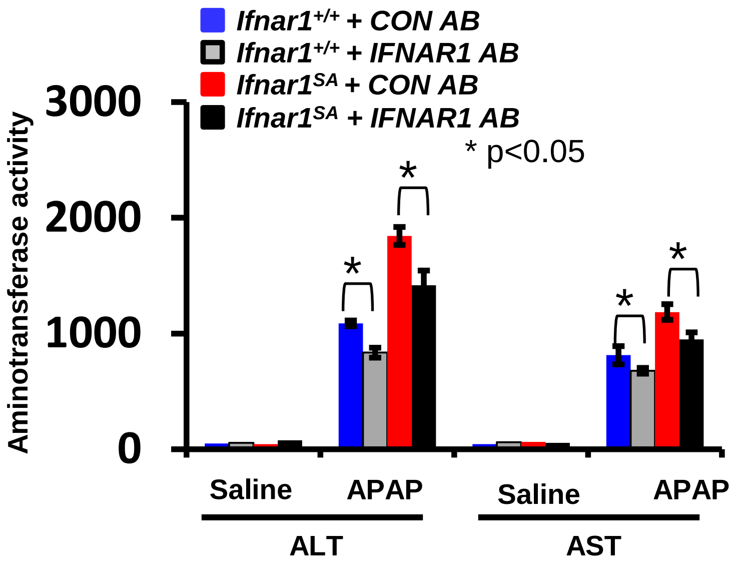

Figure S21: AST and ALT activity in plasma from *Ifnar1*$^{+}$ or *Ifnar1*^{SA} mice (n=3 each) collected 24 h after the injection of acetaminophen (APAP, 150mg/kg, i.p.). Where indicated, mice were also injected with control or IFNAR1 antibody (100µg) at 0, 8 and 16h after APAP treatment. *: $p<0.05$
