# Supplementary material for: Triggering ubiquitination of IFNAR1 protects tissues from inflammatory injury
Source: EMBO Mol Med. 2014 Jan 31;6(3):384–97. doi: 10.1002/emmm.201303236 (PMC3958312; doi:10.1002/emmm.201303236)
Supplement: Supplementary file 26 [file emmm0006-0384-sd26.pdf]

S22

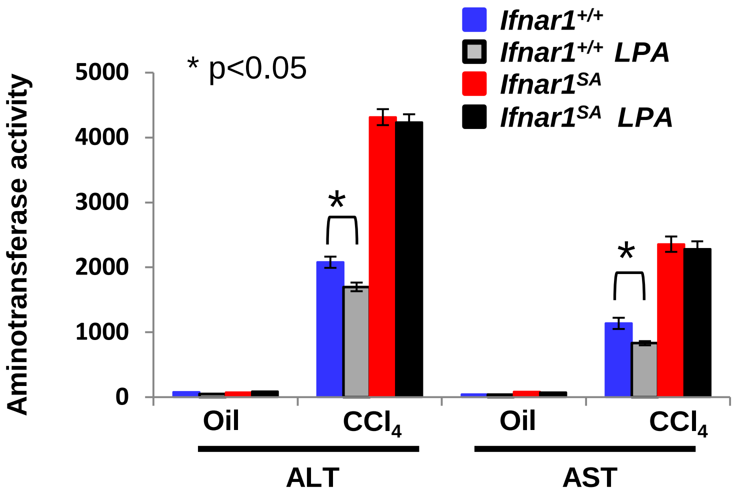

Figure S22: AST and ALT activity in plasma from *Ifnar1*$^{+}$ or *Ifnar1*^{SA} mice (n=3 each) collected 48 h after the injection of CCl_4 (0.5 ml/kg, i.p.). Where indicated, mice were also injected with LPA (10mg/kg, administered i.v. once at the time of CCl_4 treatment). *: $p<0.05$
