# Supplementary material for: Triggering ubiquitination of IFNAR1 protects tissues from inflammatory injury
Source: EMBO Mol Med. 2014 Jan 31;6(3):384–97. doi: 10.1002/emmm.201303236 (PMC3958312; doi:10.1002/emmm.201303236)
Supplement: Supplementary file 28 [file emmm0006-0384-sd28.pdf]

Supplementary Table 1: Blood cell and serum chemistry as well as tissue mitochondrial function analyses in six-eight week old female wild type and *Ifnar1*^{SA} mice

Parameter	***WT (Mean)***	***SA/SA (Mean)***	***P***	***Normal range***
**Blood cell count**				
Red blood cells (10^6/µl)	8.9-10.7 (9.4)	8.5-9.7 (9.1)	0.11	5.5-11.0
White blood cells (10^3/µl)	4.5-9.3 (7.1)	2.2-6.8 (4.7)	0.74	5.5-10.5
Haemoglobin (g/dL)	12.3-14.9 (13.6)	12.6-13.9 (13.0)	0.21	
Haemocrit (%)	41-47 (42)	39-43 (40)	0.17	
MCV, fL	43-46 (45)	42-46 (44)	1.00	
MCH, pG	13.9-15.1 (14.5)	13.7-14.9 (14.3)	0.30	
MCHC, g/dL	31.9-32.5 (32.3)	31.8-33.0 (32.3)	0.30	
Red Cell Distribution Width (%)	14.2-15.4 (14.8)	14.5-16.7 (15.0)	0.40	
Neutrophils (10^3/µL)	0.22-0.74 (0.79)	0.24-1.37 (0.68)	0.80	
Lymphocytes (10^3/µL)	4.13-8.23 (6.23)	1.81-6.29 (3.32)	0.14	
Eosinophils (10^3/µL)	0.09-1.14 (0.09)	0.02-0.07 (0.04)	0.24	
**Blood chemistry**				
Glucose (mg/dL)	80-320 (141)	38-194 (98)	0.134	90-192
Blood Urea Nitrogen (mg/dL)	19-31 (22)	15-55 (25)	0.74	18-29
Creatine (mg/dL)	0.1-0.4 (0.2)	0.1-0.2 (0.2)	0.37	0.2-0.8
Phosphorus (mg/dL)	6.2-21.0 (11.6)	6.9-14.0 (9.4)	0.63	6.1-10.1
Calcium (mg/dL)	4.7-8.6 (6.1)	5.0-10.1 (6.7)	0.64	5.9-9.4
Sodium (mmol/L)	122-190 (151)	148-152 (149)	0.74	124-174
Pottasium (mmol/L)	2.7-9.8 (6.6)	3.6-9.3 (5.9)	0.8	4.6-8
Chloride (mmol/L)	122-152 (125)	112-124 (119)	0.87	92-120
Protein (g/dL)	3.9-7.3 (5.3)	4.9-6.3 (5.7)	0.39	3.6-6.6
Albumin (g/dL)	1.2-2.1 (1.6)	1.6-2.3 (1.9)	0.06	2.5-4.8
Globulin (g/dL)	2.2-4.7 (3.3)	2.6-4.6 (3.8)	0.15	0-6
AST (U/L)	104-251 (153)	80-115 (97)	0.76	59-247
ALT (U/L)	47-148 (85)	79-104 (91)	0.106	28-132
Bilirubin (mg/dL)	0.4-2.4 (1.1)	1.1-2.2 (1.5)	0.26	0.1-0.9
Alkaline Phosphatase (U/L)	65-158 (96)	46-205 (121)	0.54	62-209
Cholesterol (mg/dL)	54-136 (118)	77-135 (123)	0.11	36-96
Creatine Kinase (U/L)	278-366 (351)	217-471 (388)	0.68	68-1070
Mitochondrial function parameters				
Co-I, pancreas (nmoles/min/mg)	149-206 (179)	166-180 (173)	0.68	
Co-I, liver (nmoles/min/mg)	354-375 (372)	357-375 (366)	0.70	
CytOx, pancreas (µmoles/min/mg)	139-150 (145)	138-161 (149)	0.52	
CytOx, liver (µmoles/min/mg)	346-365 (356)	377-391 (375)	0.12	

MCV, Mean corpuscular volume; MCH, Mean Corpuscular Haemoglobin; MCHC, Mean Corpuscular Haemoglobin Concentration; Co-I, complex I activity; CytOx, cytochrome oxidase activity
