# Supplementary material for: Triggering ubiquitination of IFNAR1 protects tissues from inflammatory injury
Source: EMBO Mol Med. 2014 Jan 31;6(3):384–97. doi: 10.1002/emmm.201303236 (PMC3958312; doi:10.1002/emmm.201303236)
Supplement: Supplementary file 29 [file emmm0006-0384-sd29.pdf]

Supplemental Methods:

***Ifnar1^{SA}* mice:** We have previously described the generation of these knock-in mice (Zheng et al., 2011) from the C57Bl/6 ES cells previously targeted with the knock-in vector (described in (Liu et al., 2009)) and then treated with Cre to remove the neo marker in vitro. Whereas cells from heterozygous mice (*Ifnar1$^{+/SA}$*) were used in previous publications (Qian et al., 2011; Zheng et al., 2011), all of our current experiments were carried out using animals/cells that had both wild type alleles replaced by homozygous *Ifnar1^{SA}*.

Antibodies and other reagents were purchased from commercial sources including Sigma (Acetaminophen, Concanavalin A, caerulein, LPS, carbon tetrachloride), Cayman Chemicals (LPA), Biosource (anti-phospho-Ser710-PKD2), Bethyl Laboratories (anti-PKD2), Biolegend (anti-CD11b (101201), anti-CD11b-FITC (101205), anti-CD45-APC-Cy7 (103115), anti- F4/80 (123101), mouse IgG1-biotin (400103), and anti-Ly6G (127601)), eBioscience (anti-CD16/32 (14-0161), anti-CD45-FITC (11-0451), Streptavidin-PE (12-4317), Streptavidin-Dylight 594 (405222), ELISA kits for detection of IL6 (88-7064), IL1β (88-7013), TNFα (88-7324), and Fixable Viability Dye eFluor®506 (65-0866)), PBL Interferon Source Inc (VeriKine-HS™ Mouse IFNβ serum ELISA kit (42410)), Cell Signaling (anti-pSTAT1 (9167), anti-p38 (8690), anti-phospho-p38 (4631)), Leinco Technologies, Inc (anti-IFNAR1 (I-402)), Invitrogen (Trizol, ProLong gold antifade reagent, anti-rat AF488 (A-11006) and anti-rabbit AF488 (A11070) and normal rat IgG (A10700)), Bioassay Systems (QuantiChrom a-Amylase Assay Kit, DAMY-100), Sakura Finetek USA, Inc (Tissue-Tek O.C.T. compound (117-10)), APP Pharmaceuticals (5-Fluorouracil (117-10)) and Santa Cruz (anti-ubiquitin, (sc-8017)). Anti-phospho-Ser526/Ser535 rabbit polyclonal antibody was previously described (Kumar et al., 2004).

Immunohistochemistry and cytokine measurement: Pancreata harvested from mice were frozen in Tissue-Tek O.C.T. compound and cryo-sectioned using the Leica CM3050 S Cryostats, fixed in acetone, washed and blocked with PBS containing 5% goat serum. The sections were incubated with primary antibodies for 1h, washed and incubated with Alexa Fluor 488 goat anti-Rat antibodies for 1h. Co-immunofluorescence studies were carried out by incubating slides with anti-CD11b-FITC or anti-CD45-FITC followed by biotinylated anti-F4/80 antibodies. The slides were then incubated with DyLight 594-conjugated streptavidin. The sections were then washed and incubated with DAPI for 2 min and mounted with cover slip in ProLong gold antifade reagent. TNFα, IL1β, IFNβ and IL6 levels in plasma harvested from mice following inflammatory challenges were determined using ELISA kits (eBioscience or PBL Interferon Source) following manufacturer's recommendations. Cytokine levels in plasma of mice induced with acute pancreatitis were analyzed by Luminex technology using the Milliplex Mouse Cytokine-Chemokine panel at the Luminex Core Facility of the University of Pittsburgh Cancer Institute. Immunoprecipitation and immunoblotting was carried out as described previously (Bhattacharya et al., 2011).

Flow cytometry: Blood was stained with biotinylated antibodies against mouse IFNAR1 or biotinylated isotype control antibodies. Red blood cells were lysed using the RBC lysis buffer (155mM NH_4Cl, 12mM $KHCO_3$, and 0.1mM EDTA) for 5 minutes and remaining leukocytes were washed with PBS and analyzed as outlined below. Peritoneal cells were stained with viability dye, washed and stained with a cocktail

containing APC-Cy7 labeled antibodies against anti-CD45, biotinylated antibodies against mouse IFNAR1 or biotinylated isotype control antibodies. Cells were then washed and stained with phycoerythrin conjugated streptavidin. Splenocytes (as a single cell suspension) were first incubated with red blood cell lysis buffer, washed and then fixed with 4% paraformaldehyde. For pSTAT1 staining cells were permeabilized with 90% methanol and incubated with primary antibodies against pSTAT1 followed by anti-rabbit AF488. All cells were analyzed using LSRFortessa flow cytometer (BD Biosciences).

Real time PCR: Pancreas and liver tissues were flash-frozen and pulverized in liquid nitrogen, homogenized in Trizol reagent, and extracted with chloroform. Reverse transcription was carried out using Revertaid first strand cDNA synthesis kit (K1621, Thermo Scientific) and the cDNA was used for real time QPCR analysis of expression of cytokine and β-actin mRNA using the following primers: TNFα (FW, 5'-CATCTTCTCAAAATTCGAGTGACAA-3', REV, 5'-TGGGAGTAGACAAGGTACAACCC-3'), IL-10 (FW, 5'-GCTCTTGCACTACCAAAGCC-3', REV, 5'-CTGCTGATCCTCATGCCAGT-3', CXCL1 (FW, 5'-ACCGAAGTCATAGCCACACT-3', REV, 5'-GTGCCATCAGAGCAGTCTGT-3'), CCL2(FW, 5'-CTGTAGTTTTTGTCACCAAGCTCA-3', REV, 5'-GTGCTGAAGACCTTAGGGCA-3'), CCL5 (FW, 5'-CCTCACCATATGGCTCGGAC-3', REV, 5'-ACGACTGCAAGATTGGAGCA-3'), ISG15 (FW, 5'-GGAACGAAAGGGGCCACAGCA-3', REV, 5'-CCTCCATGGGCCTTCCCTCGA-3'), IRF7 (FW, 5'-CCACACCCCCATCTTCGA-3', REV, 5'-CCTCCGAGCCCGAAACTC-3'), STAT1 (FW, 5'-CGCGCATGCAACTGGCATATAACT-3', REV, 5'-AAGCTCGAACCACTGTGACATCCT-3') and IFNAR1 (FW, 5'-ACACCCTAAAGTGGAGCAGC-3', REV, 5'-TTGTCCGGGAGGAGAGATGT-3') using Applied biosystems 7500 Fast real time PCR system.

Mitochondrial preparation and assays: Mitochondria were prepared by differential centrifugation as described previously (Prabu et al., 2006). Assays for complex I and cytochrome oxidase were carried out as described (Birch-Machin and Turnbull, 2001) using a Cary UV-Vis Spectrophotometer. Briefly, complex I activity (NADH:ubiquinone oxidoreductase) was measured by incubating 50 µg of freeze-thawed mitochondrial extract in 1ml of assay medium (25mM potassium phosphate, pH 7.4, 5mM magnesium chloride, 2mM sodium cyanide, 2.5mg/ml bovine serum albumin, 13mM NADH, 65µM ubiquinone and 2µg/ml antimycin A) and measuring the decrease in absorbance at 340 nm due to NADH oxidation. Cytochrome oxidase activity was measured by incubating 5µg of freeze-thawed mitochondrial extract in 1mL of assay medium (25mM potassium phosphate, pH 7.4, 0.45mM dodecyl maltoside and 15mM reduced cytochrome c) and measuring the decrease in absorbance at 550 nm due to cytochrome c oxidation.

References

Bhattacharya, S., Qian, J., Tzimas, C., Baker, D. P., Koumenis, C., Diehl, J. A., and Fuchs, S. Y. (2011). Role of p38 protein kinase in the ligand-independent ubiquitination and down-regulation of the IFNAR1 chain of type I interferon receptor. J Biol Chem *286*, 22069-22076.

Birch-Machin, M. A., and Turnbull, D. M. (2001). Assaying mitochondrial respiratory complex activity in mitochondria isolated from human cells and tissues. Methods Cell Biol *65*, 97-117.

Kumar, K. G., Krolewski, J. J., and Fuchs, S. Y. (2004). Phosphorylation and specific ubiquitin acceptor sites are required for ubiquitination and degradation of the IFNAR1 subunit of type I interferon receptor. J Biol Chem *279*, 46614-46620.

Liu, J., HuangFu, W. C., Kumar, K. G., Qian, J., Casey, J. P., Hamanaka, R. B., Grigoriadou, C., Aldabe, R., Diehl, J. A., and Fuchs, S. Y. (2009). Virus-induced unfolded protein response attenuates antiviral defenses via phosphorylation-dependent degradation of the type I interferon receptor. Cell Host Microbe *5*, 72-83.
Prabu, S. K., Anandatheerthavarada, H. K., Raza, H., Srinivasan, S., Spear, J. F., and Avadhani, N. G. (2006). Protein kinase A-mediated phosphorylation modulates cytochrome c oxidase function and augments hypoxia and myocardial ischemia-related injury. J Biol Chem *281*, 2061-2070.
Qian, J., Zheng, H., Huangfu, W. C., Liu, J., Carbone, C. J., Leu, N. A., Baker, D. P., and Fuchs, S. Y. (2011). Pathogen recognition receptor signaling accelerates phosphorylation-dependent degradation of IFNAR1. PLoS Pathog *7*, e1002065.
Zheng, H., Qian, J., Carbone, C. J., Leu, N. A., Baker, D. P., and Fuchs, S. Y. (2011). Vascular endothelial growth factor-induced elimination of the type 1 interferon receptor is required for efficient angiogenesis. Blood *118*, 4003-4006.
