# Supplementary material for: Triggering ubiquitination of IFNAR1 protects tissues from inflammatory injury
Source: EMBO Mol Med. 2014 Jan 31;6(3):384–97. doi: 10.1002/emmm.201303236 (PMC3958312; doi:10.1002/emmm.201303236)
Supplement: Supplementary file 30 [file emmm0006-0384-sd30.pdf]

Bhattacharya et al, Supporting information

Table of Content

1. Supporting Information Figure 1: ELISA analyses of the levels of IFNβ
2. Supporting Information Figure 2: Fold induction of mRNA of *ISGs*
3. Supporting Information Figure 3: H&E staining of pancreata of mice with acute pancreatitis.
4. Supporting Information Figure 4: High magnification of H&E staining of pancreata of mice with acute pancreatitis.
5. Supporting Information Figure 5: Quantification of acinar tissue damage.
6. Supporting Information Figure 6: FACS analysis of IFNAR1 cell surface levels in splenocytes treated with trypsin.
7. Supporting Information Figure 7: Body weight (g) and relative organ weight of $ifnar^{+/+}$ and $ifnar1^{SA}$.
8. Supporting Information Figure 8: Characteristics of splenic cells from $Ifnar1^{SA}$ mice
9. Supporting Information Figure 9: ELISA analyses of the levels of IFNβ in blood plasma of mice treated with LPS.
10. Supporting Information Figure 10: Immunofluorescence analysis of adjacent serial cryosections from spleens of mice.
11. Supporting Information Figure 11: Relative intensity of immunofluorescence from pancreatic tissues of mice with acute pancreatitis.
12. Supporting Information Figure 12: H&E staining of pancreata from mice at 4 weeks of continuous chronic caerulein treatment.
13. Supporting Information Figure 13: Fold induction of mRNA of *IFNAR1,* in liver of mice treated with compounds inducing hepatitis.
14. Supporting Information Figure 14: Relative levels of *Tnfα* mRNA in liver tissues.
15. Supporting Information Figure 15: ELISA measurement of the levels of plasma IFNβ in mice treated with $CCl_{4.}$
16. Supporting Information Figure 16: H&E staining of liver tissues.
17. Supporting Information Figure 17: H&E staining of liver tissues.
18. Supporting Information Figure 18: H&E staining of pancreata from chimeric mice.
19. Supporting Information Figure 19: H&E staining of liver tissues obtained from chimeric mice.
20. Supporting Information Figure 20: AST and ALT activity in plasma from $Ifnar1^{+}$ or $Ifnar1^{SA}$ mice.
21. Supporting Information Figure 21: AST and ALT activity in plasma from $Ifnar1^{+}$ or $Ifnar1^{SA}$ mice.
22. Supporting Information Figure 22: AST and ALT activity in plasma from $Ifnar1^{+}$ or $Ifnar1^{SA}$ mice.
23. Supporting Information Figure 23: AST and ALT activity in plasma from $Ifnar1^{+}$ or $Ifnar1^{SA}$ mice.
24. Supporting Information Table 1
25. Supporting Information Methods
